# Supplementary material for: Triterpenoids from Kochiae Fructus: Glucose Uptake in 3T3-L1 Adipocytes and α-Glucosidase Inhibition, In Silico Molecular Docking
Source: Int J Mol Sci. 2023 Jan 26;24(3):2454. doi: 10.3390/ijms24032454 (PMC9916857; doi:10.3390/ijms24032454)
Supplement: Supplementary file 1 [file ijms-24-02454-s001.zip › ijms-2110485-supplementary.pdf]

International Journal of *Molecular Sciences*

MDPI

Supplementary Materials

Triterpenoids from Kochiae Fructus: Glucose Uptake in 3T3-L1 Adipocytes and α-Glucosidase Inhibition, in Silico Molecular Docking

Xue-Lin Chen [1,2,†], Kun Zhang [1,2,†], Xia Zhao [1], Han-Lei Wang [1,2], Mei Han [1,2], Ru Li [1,2], Zhen-Nan Zhang [1,2] and Yu-Mei Zhang [1,2,*]

1 Key Laboratory of Tropical Plant Resource and Sustainable Use, Xishuangbanna Tropical Botanical Garden, Chinese Academy of Sciences, Kunming 650223, China; chenxuelin@xtbg.ac.cn (X.-L.C.); zhangkun@xtbg.ac.cn (K.Z.); zhaoxia@xtbg.ac.cn (X.Z.); wanghanlei@xtbg.ac.cn (H.-L.W.); hanmei@xtbg.ac.cn (M.H.); liru@xtbg.ac.cn (R.L.); zhangzhennan@xtbg.ac.cn (Z.-N.Z.)
2 School of Life Science, University of Chinese Academy of Sciences, Beijing 100049, China
* Correspondence: zymei@xtbg.ac.cn; Tel.: +86-871-6511-2766
† These authors contributed equally to this work.

Figure S1. ^{1}H NMR spectrum of compound **1** (methanol-d_4, 500MHz).
Figure S2. ^{13}C NMR spectrum of compound **1** (methanol-d_4, 125MHz).
Figure S3. HSQC spectrum of compound **1.**
Figure S4. HMBC spectrum of compound **1.**
Figure S5. ^{1}H-^{1}H COSY spectrum of compound **1.**
Figure S6. ROESY spectrum of compound **1**.
Figure S7. HSQC-TOCSY spectrum of compound **1**.
Figure S8. HRESI-MS spectra of compound **1**.
Figure S9. Optical rotations spectrum of compound **1**.
Figure S10. UV spectrum of compound **1**.
Figure S11. IR spectrum of compound **1**.
Figure S12. ^{1}H NMR spectrum of compound **2** (methanol-d_4, 500MHz).
Figure S13. ^{13}C NMR spectrum of compound **2** (methanol-d_4, 125MHz).
Figure S14. HSQC spectrum of compound **2.**
Figure S15. HMBC spectrum of compound **2.**
Figure S16. ^{1}H-^{1}H COSY spectrum of compound **2.**
Figure S17. ROESY spectrum of compound **2**.
Figure S18. HSQC-TOCSY spectrum of compound **2**.
Figure S19. HRESI-MS spectra of compound **2**.
Figure S20. Optical rotations spectrum of compound **2**.
Figure S21. UV spectrum of compound **2**.
Figure S22. IR spectrum of compound **2**.
Figure S23. ^{1}H NMR spectrum of compound **3** (methanol-d_4, 500MHz).
Figure S24. ^{13}C NMR spectrum of compound **3** (methanol-d_4, 125MHz).
Figure S25. HSQC spectrum of compound **3.**
Figure S26. HMBC spectrum of compound **3.**
Figure S27. ^{1}H-^{1}H COSY spectrum of compound **3.**
Figure S28. ROESY spectrum of compound **3**.
Figure S29. HRESI-MS spectra of compound **3**.
Figure S30. Optical rotations spectrum of compound **3**.
Figure S31. UV spectrum of compound **3**.
Figure S32. IR spectrum of compound **3**.
Figure S33. The CD spectra of compounds **1**, **2**, and **3** in MeOH.
Figure S34. ^{1}H NMR spectrum of compound **4** (methanol-d_4, 500MHz).
Figure S35. ^{1}H NMR spectrum of compound **5** (methanol-d_4, 500MHz).

Figure S36. ^{1}H NMR spectrum of compound **6** (methanol-d_4, 500MHz).
Figure S37. ^{1}H NMR spectrum of compound **7** (methanol-d_4, 500MHz).
Figure S38. ^{1}H NMR spectrum of compound **8** (methanol-d_4, 500MHz).
Figure S39. ^{1}H NMR spectrum of compound **9** (methanol-d_4, 500MHz).
Figure S40. ^{1}H NMR spectrum of compound **10** (methanol-d_4, 600MHz).
Figure S41. ^{1}H NMR spectrum of compound **11** (methanol-d_4, 500MHz).
Figure S42. ^{1}H NMR spectrum of compound **12** (methanol-d_4, 500MHz).
Figure S43. ^{1}H NMR spectrum of compound **13** (methanol-d_4, 600MHz).
Figure S44. ^{1}H NMR spectrum of compound **14** (methanol-d_4, 600MHz).
Figure S45. ^{1}H NMR spectrum of compound **15** (methanol-d_4, 600MHz).
Figure S46. ^{1}H NMR spectrum of compound **16** (methanol-d_4, 600MHz).
Figure S47. ^{1}H NMR spectrum of compound **17** (methanol-d_4, 600MHz).
Table S1. The data that was used to calculate the IC_{50} value for compound **3**.
Table S2. The data that was used to calculate the IC_{50} value for compound **9**.
Table S3. The data that was used to calculate the IC_{50} value for compound **13**.
Table S4. The data that was used to calculate the IC_{50} value for acarbose.
Table S5. The data that was used to calculate the Km, K_i, *and* K_i' value for compound **3**.
Table S6. The data that was used to calculate the Km, K_i, *and* K_i' value for compound **9**.
Table S7. The data that was used to calculate the Km, K_i, *and* K_i' value for compound **13**.

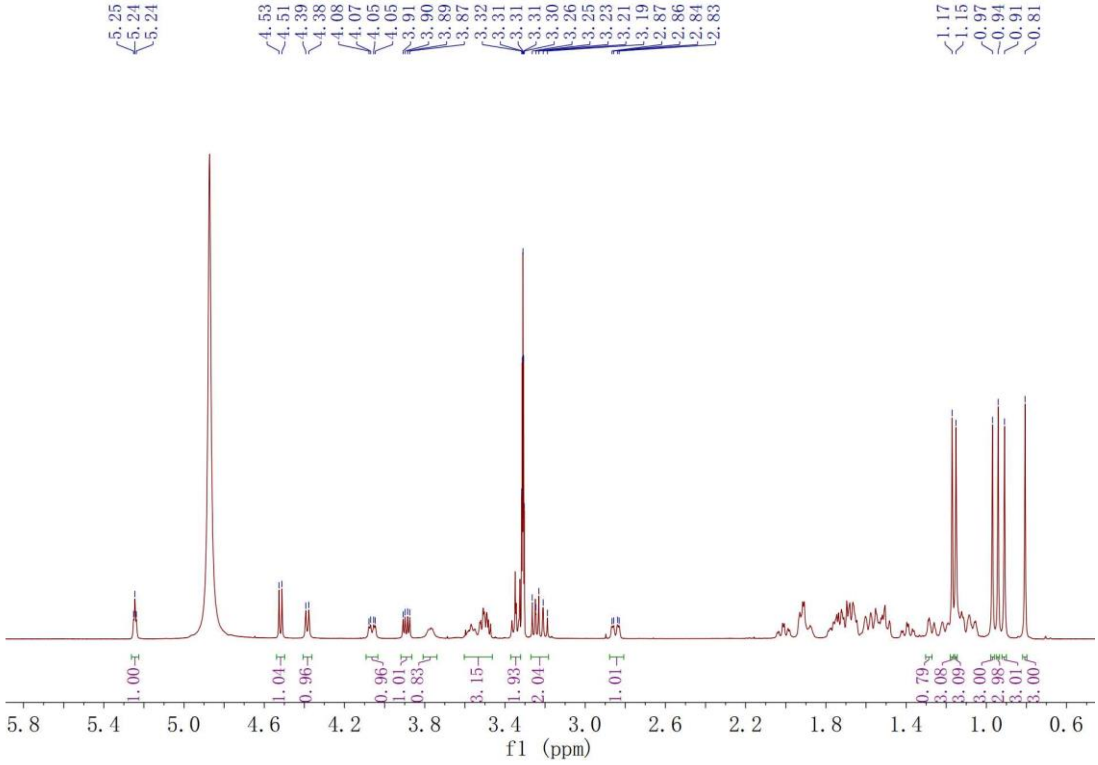

Figure S1. ^{1}H NMR spectrum of compound **1** (methanol-d_4, 500MHz).

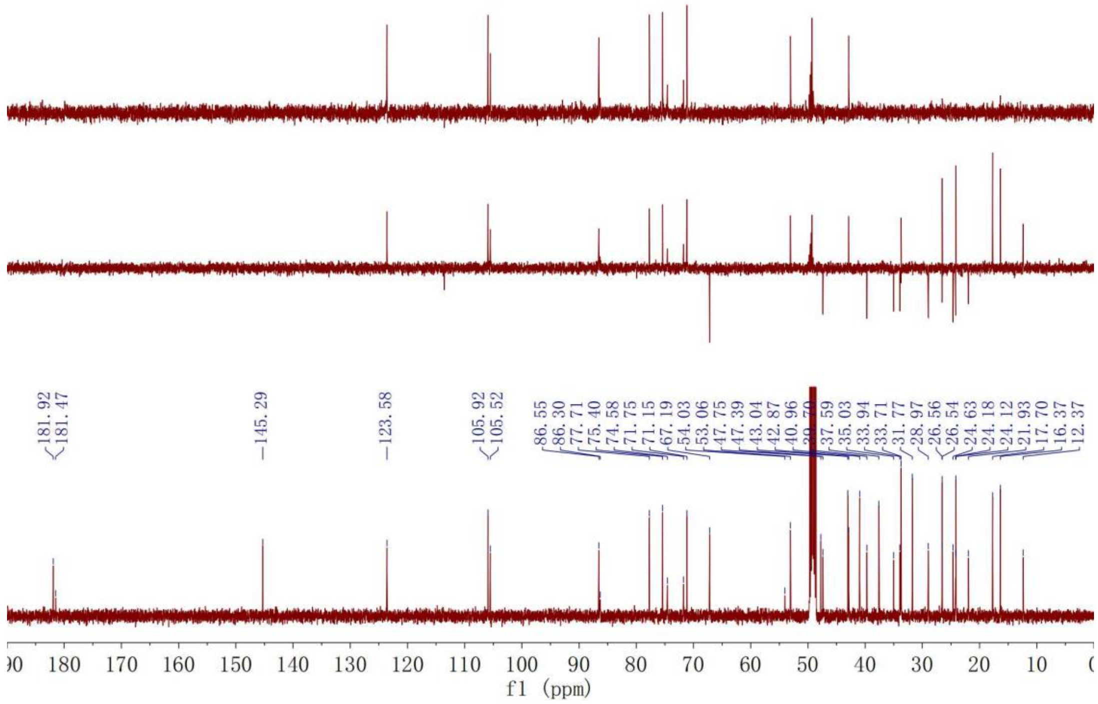

Figure S2. ^{13}C NMR spectrum of compound **1** (methanol-d_4, 125MHz).

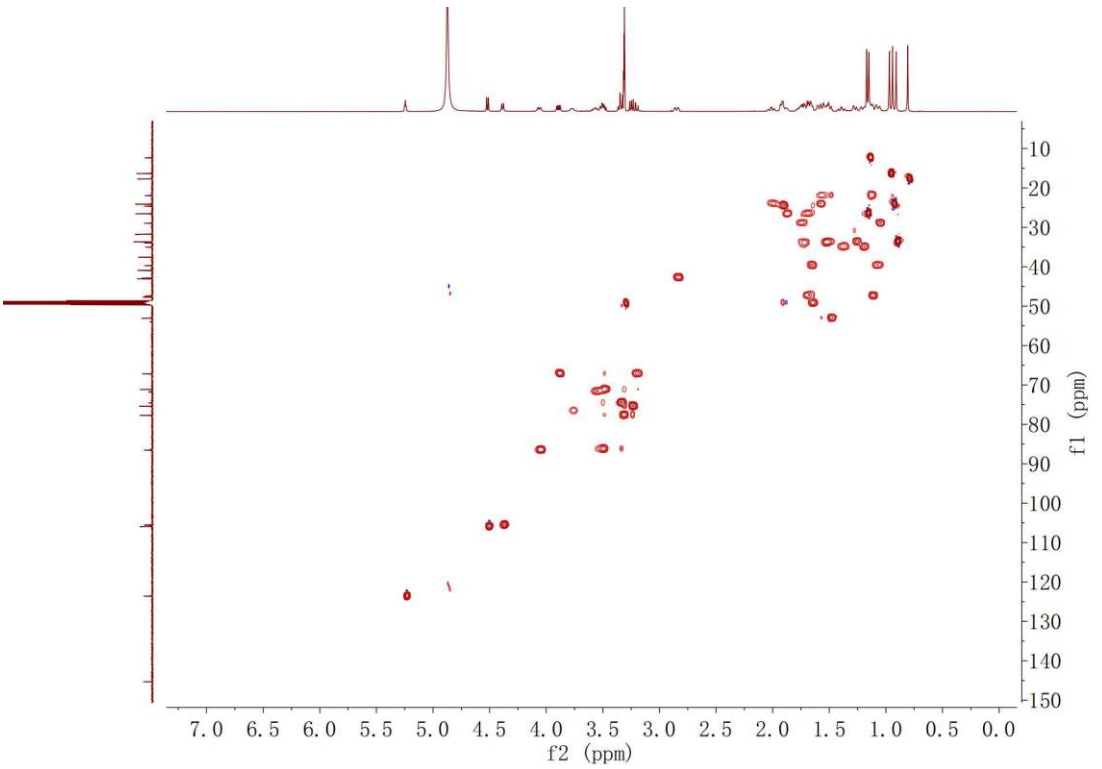

Figure S3. HSQC spectrum of compound **1.**

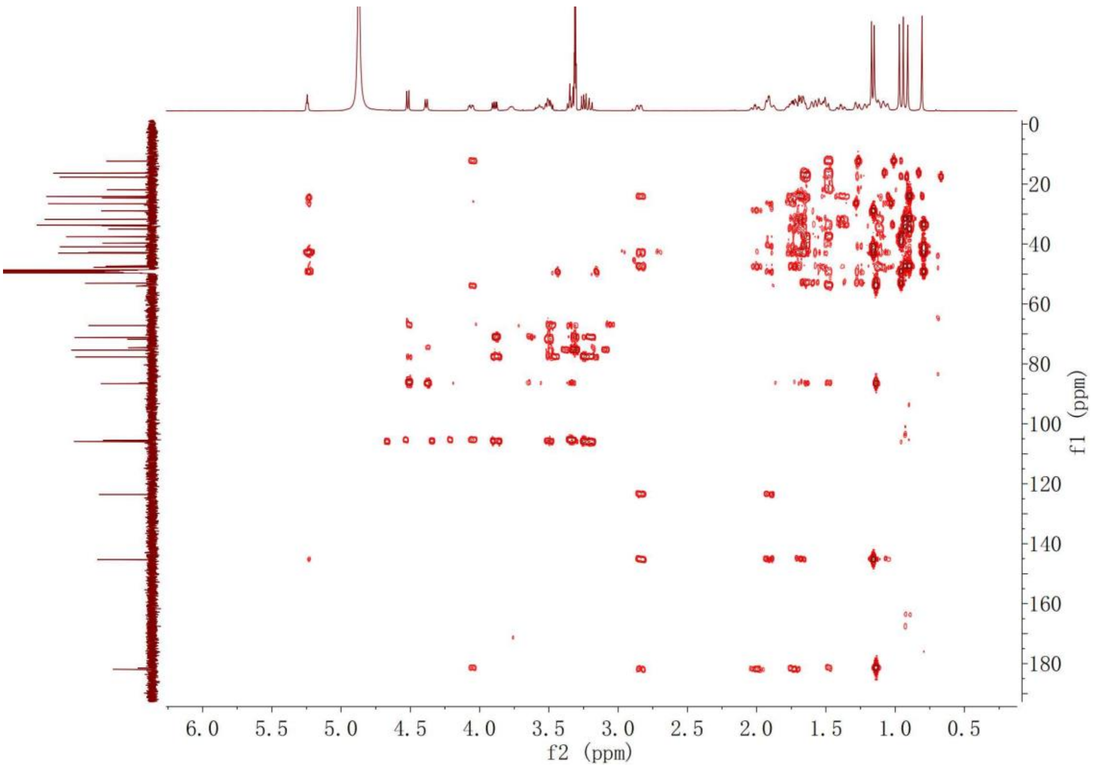

Figure S4. HMBC spectrum of compound **1.**

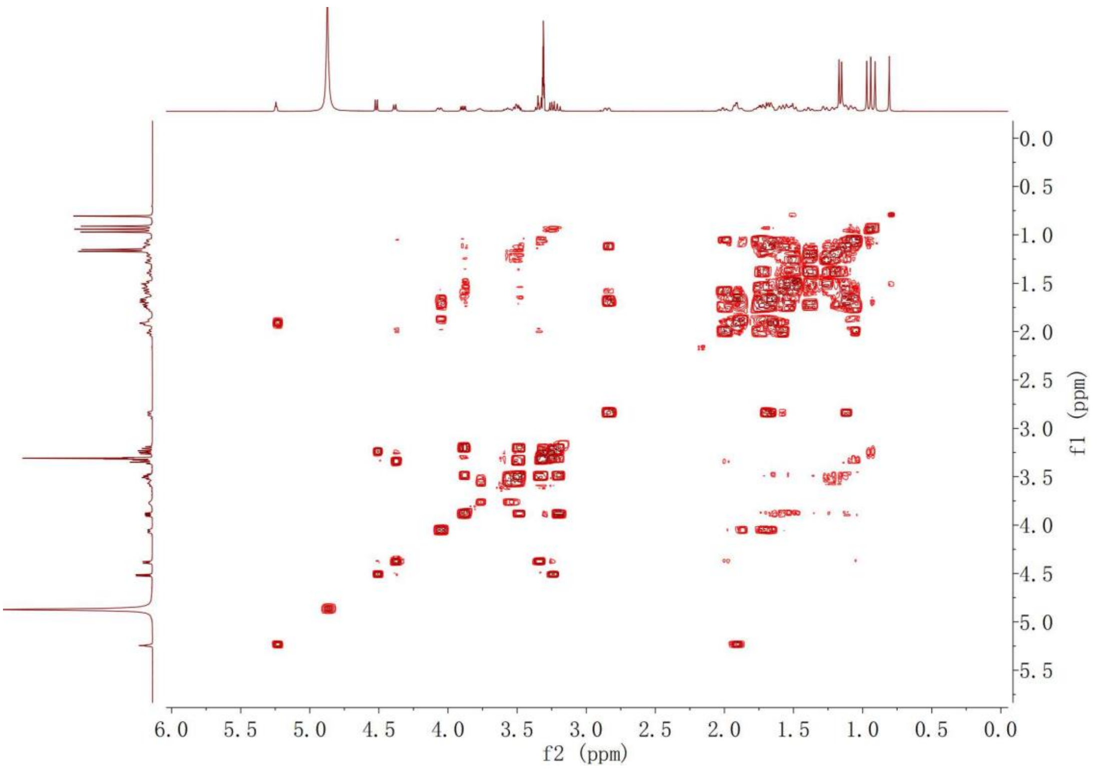

Figure S5. ^1H-^1H COSY spectrum of compound **1.**

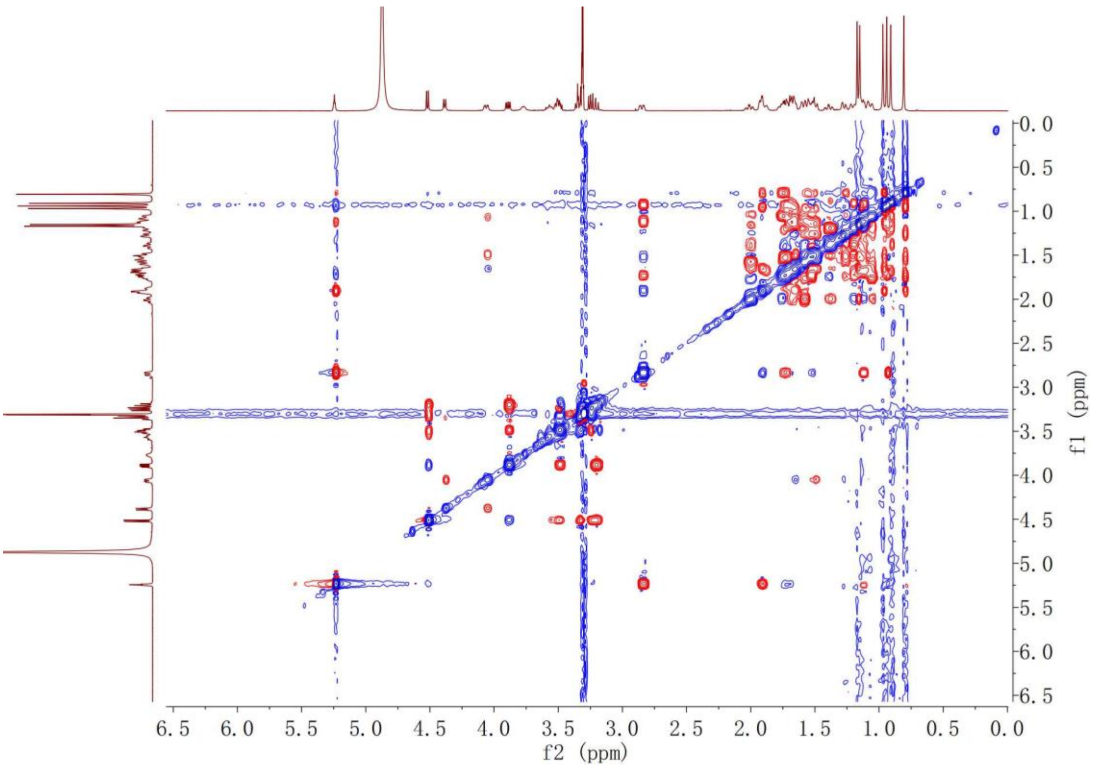

Figure S6. ROESY spectrum of compound **1**.

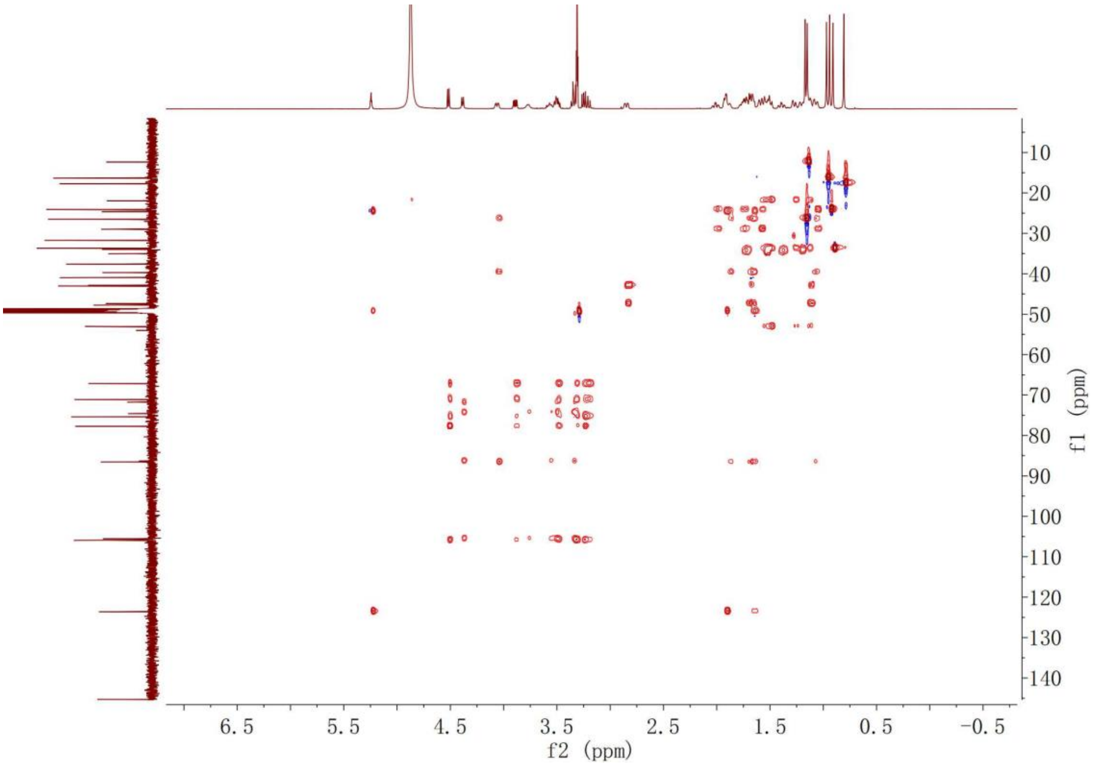

Figure S7. HSQC-TOCSY spectrum of compound **1**.

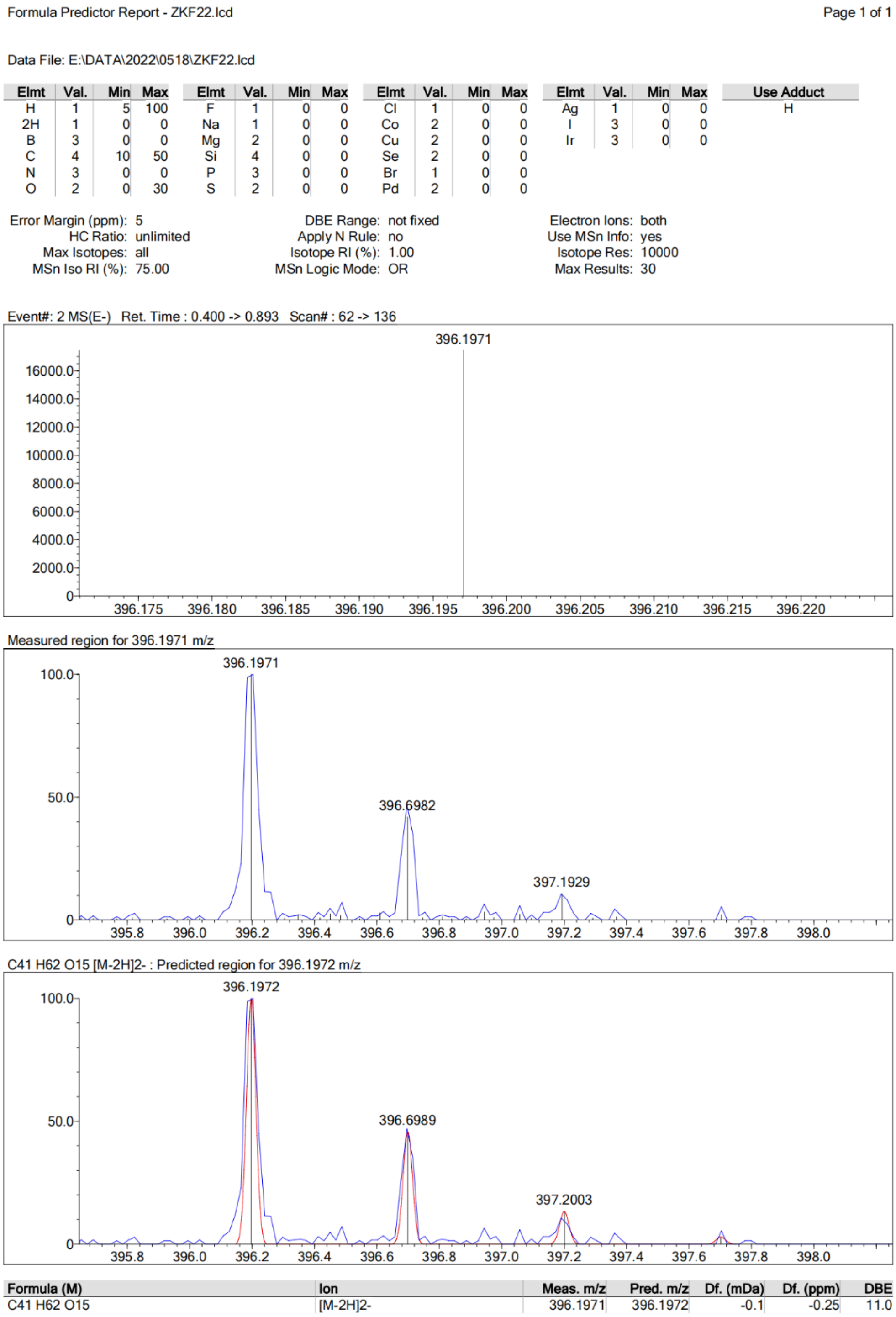

Figure S8. HRESI-MS spectra of compound **1**.

Rudolph Research Analytical

This sample was measured on an Autopol VI, Serial #91058
Manufactured by Rudolph Research Analytical, Hackettstown, NJ, USA.

Measurement Date : Tuesday, 31-MAY-2022

Set Temperature : OFF

Time Delay : Disabled

Delay between Measurement : Disabled

n	Average	Std.Dev.	% RSD	Maximum	Minimum
5	17.22	0.58	3.36	18.18	16.58

S.No	Sample ID	Time	Result	Scale	OR °Arc	WLG.nm	Lg.mm	Conc.g/100ml	Temp.
1	ZKF-22	06:55:35 PM	17.11	SR	0.032	589	100.00	0.187	25.1
2	ZKF-22	06:55:42 PM	16.58	SR	0.031	589	100.00	0.187	25.1
3	ZKF-22	06:55:48 PM	17.11	SR	0.032	589	100.00	0.187	25.1
4	ZKF-22	06:55:54 PM	17.11	SR	0.032	589	100.00	0.187	25.0
5	ZKF-22	06:56:01 PM	18.18	SR	0.034	589	100.00	0.187	25.0

Figure S9. Optical rotations spectrum of compound **1**.

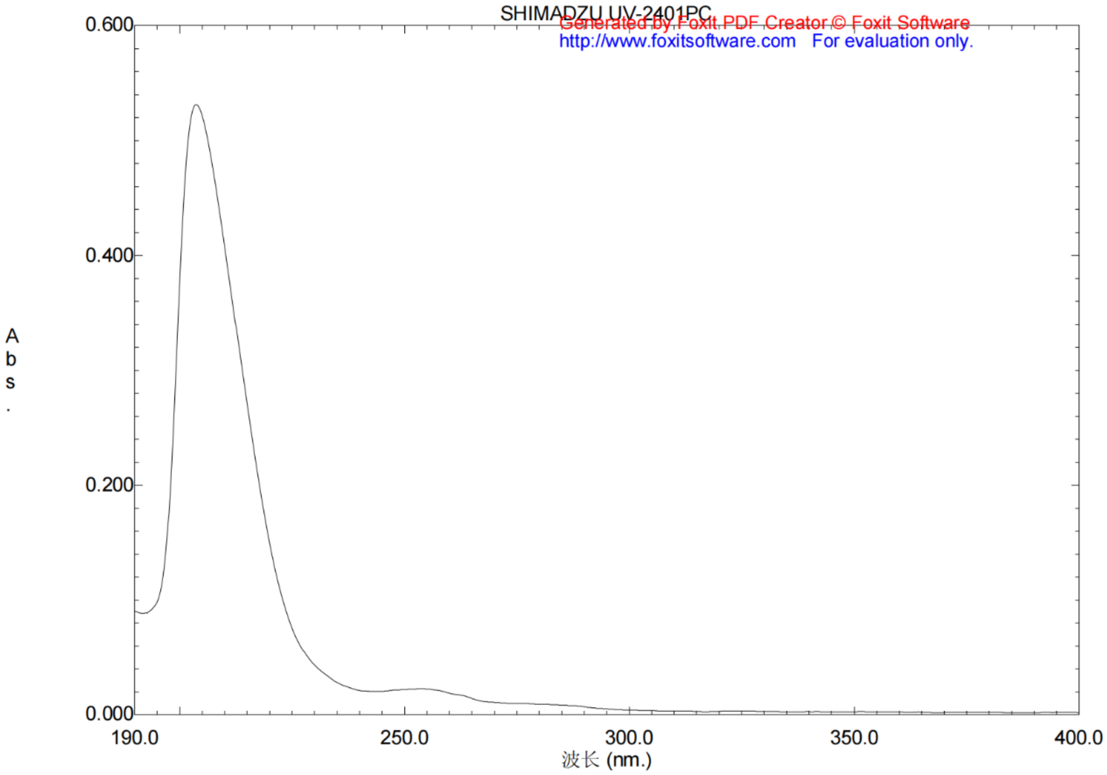

文件名: ZKF-22

ZKF-22

样品浓度：0.0748毫克/毫升
溶剂：甲醇

创建于: 20:17 22-06-01
数据: 原始

测量模式: Abs.
扫描速度: 中速
狭缝: 5.0
采样间隔: 0.2

否.	波长 (nm.)	Abs.
1	253.20	0.0224
2	203.60	0.5311

Figure S10. UV spectrum of compound **1**.

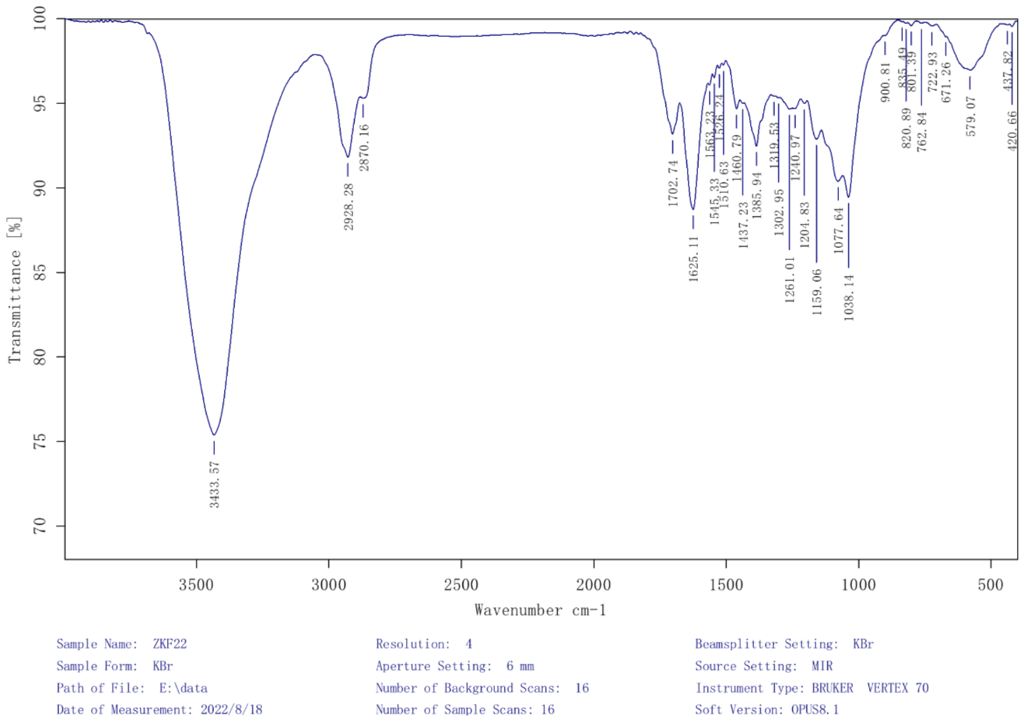

Figure S11. IR spectrum of compound **1**.

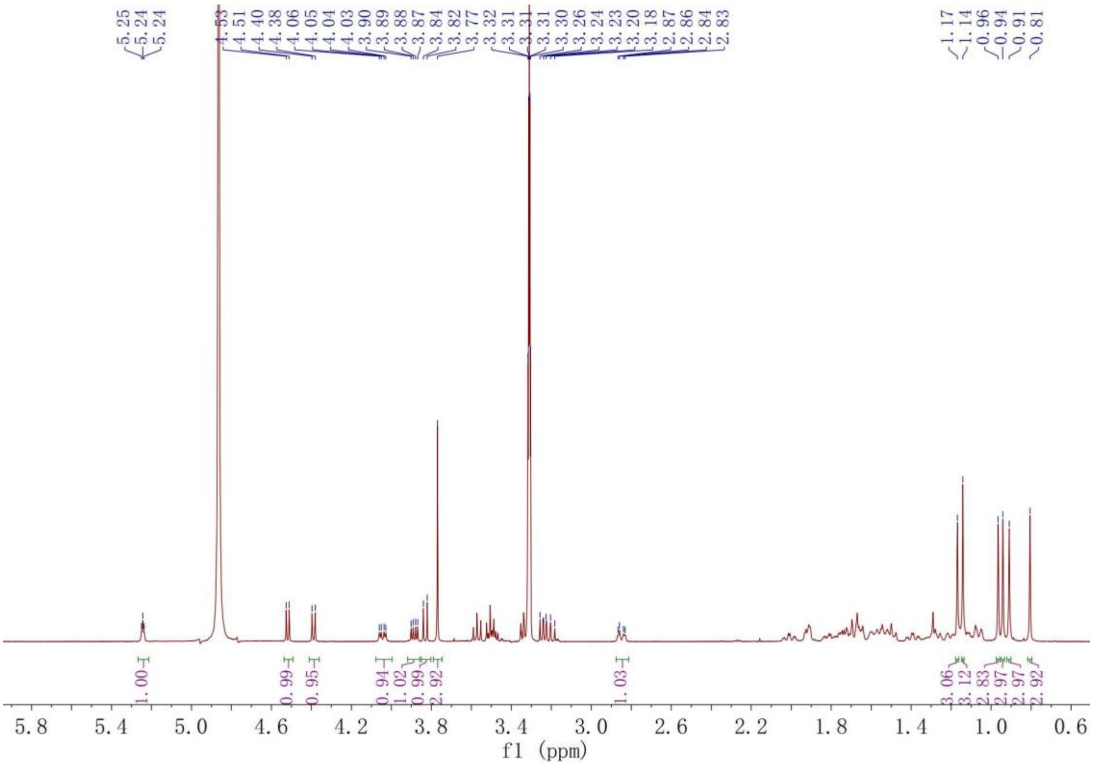

Figure S12. ^{1}H NMR spectrum of compound **2** (methanol-*d*$_4$, 500MHz).

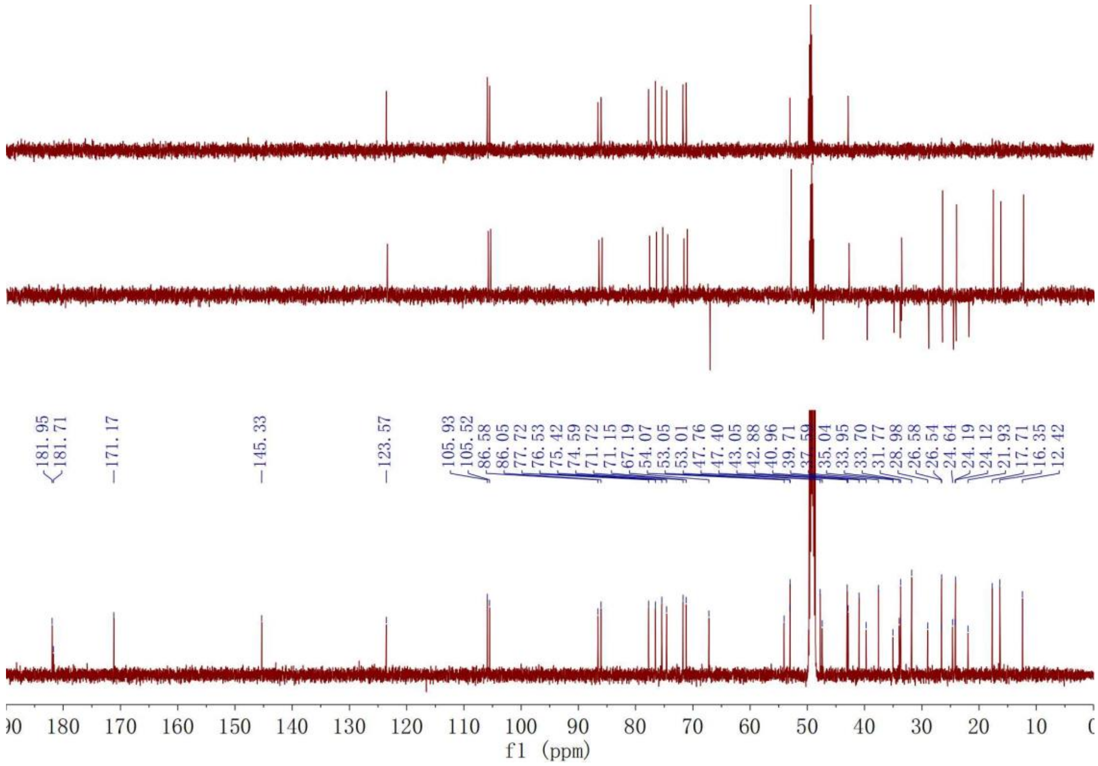

Figure S13. ^{13}C NMR spectrum of compound **2** (methanol-d_4, 125MHz).

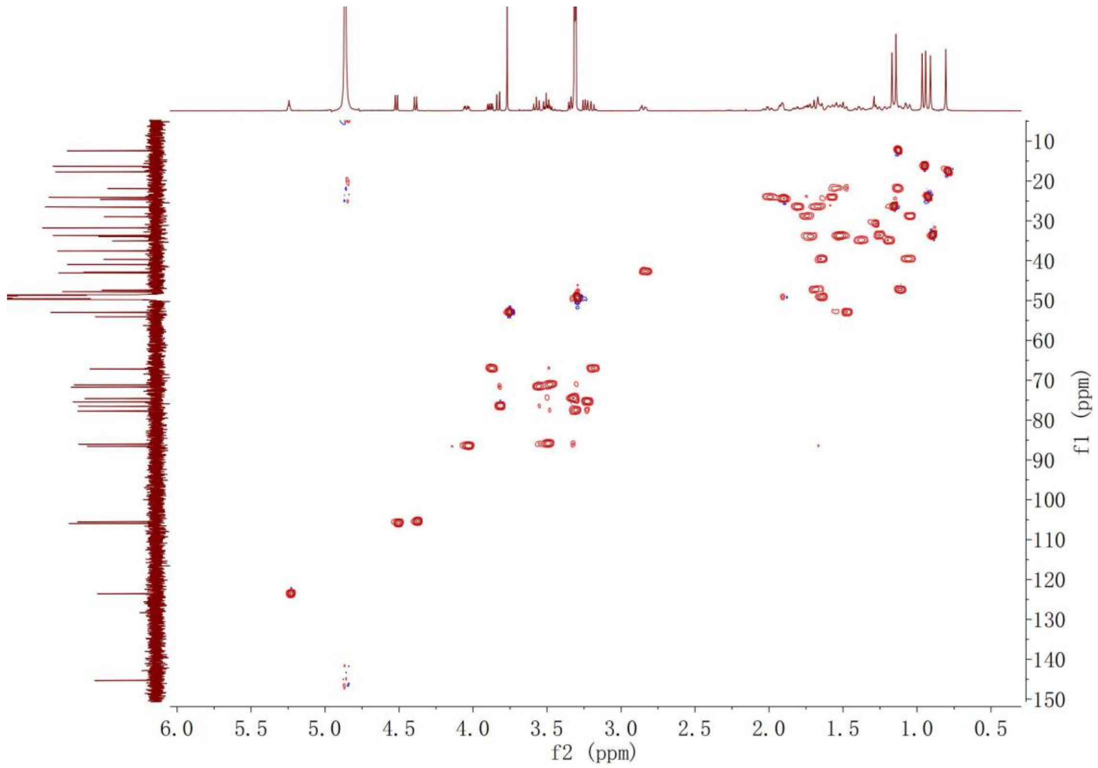

Figure S14. HSQC spectrum of compound **2.**

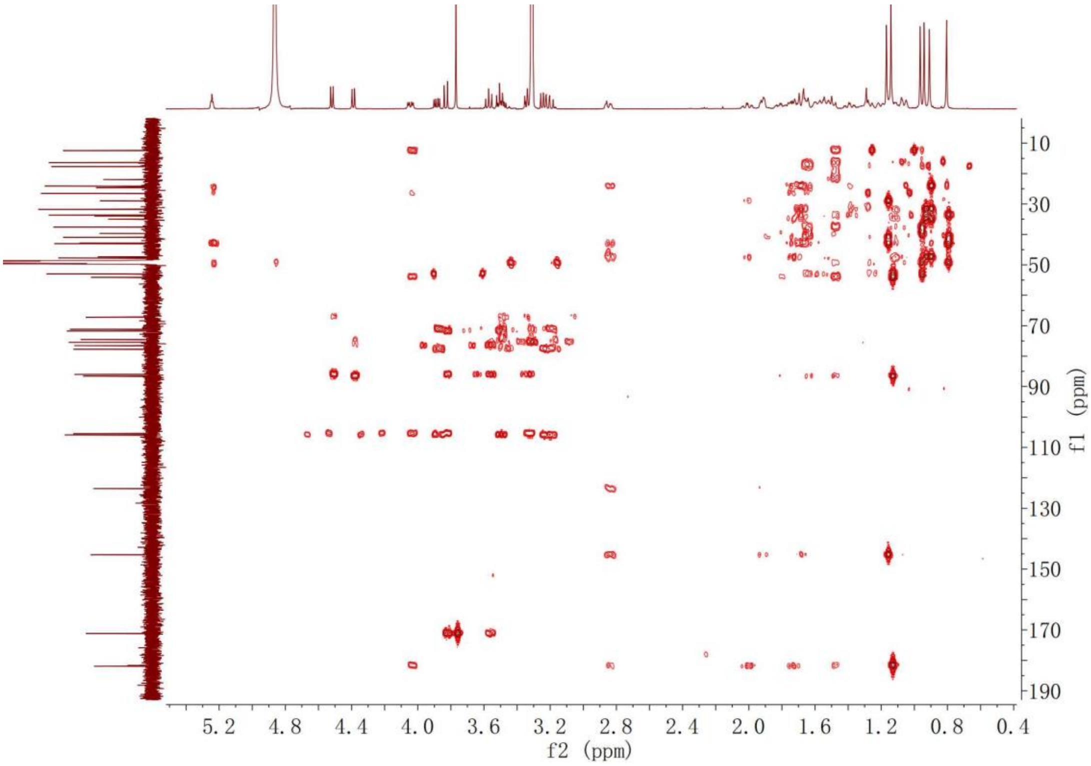

Figure S15. HMBC spectrum of compound **2.**

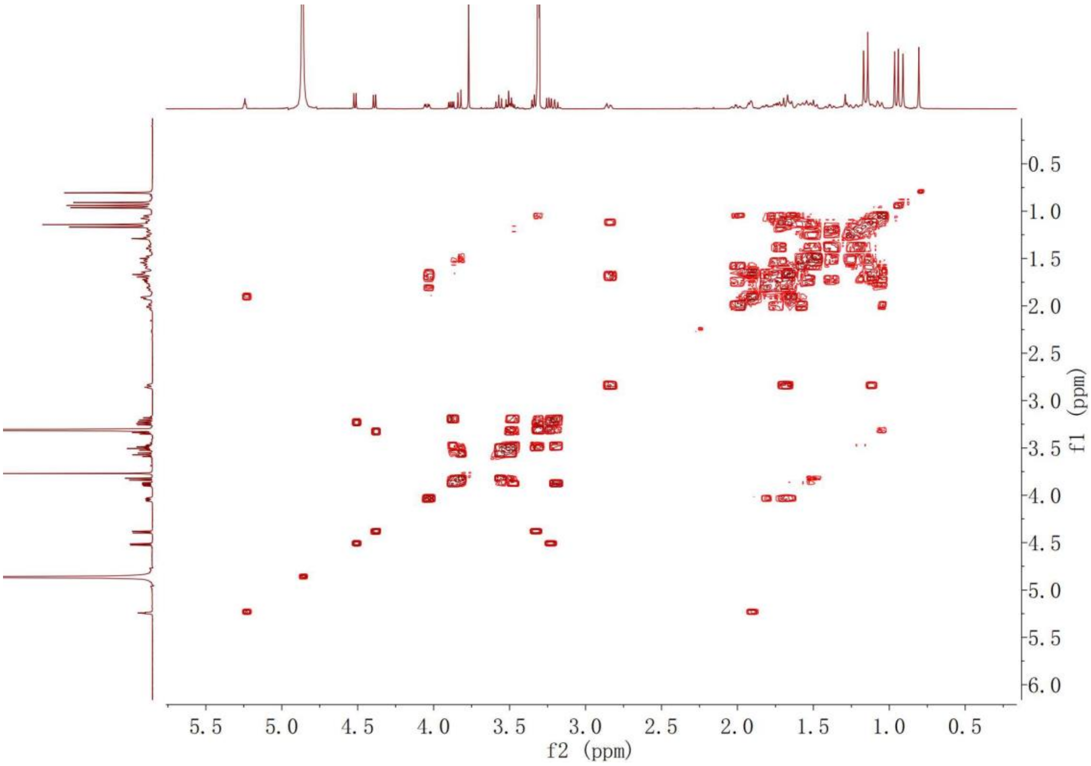

Figure S16. ^{1}H-^{1}H COSY spectrum of compound **2.**

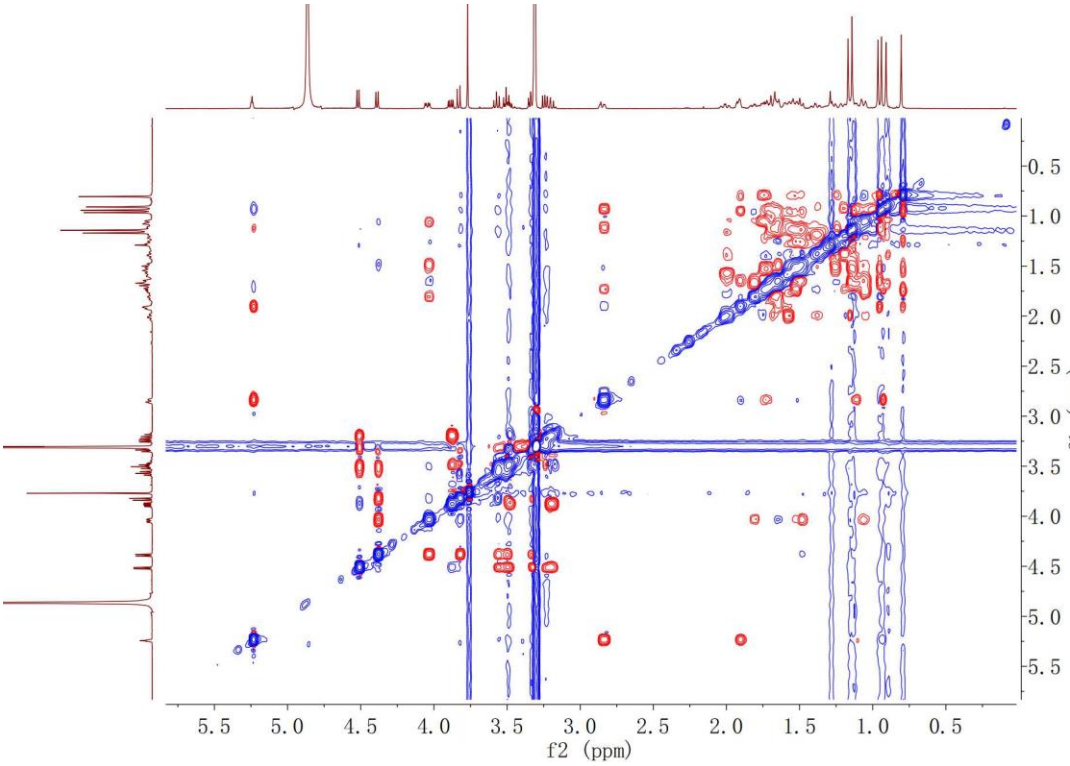

Figure S17. ROESY spectrum of compound **2**.

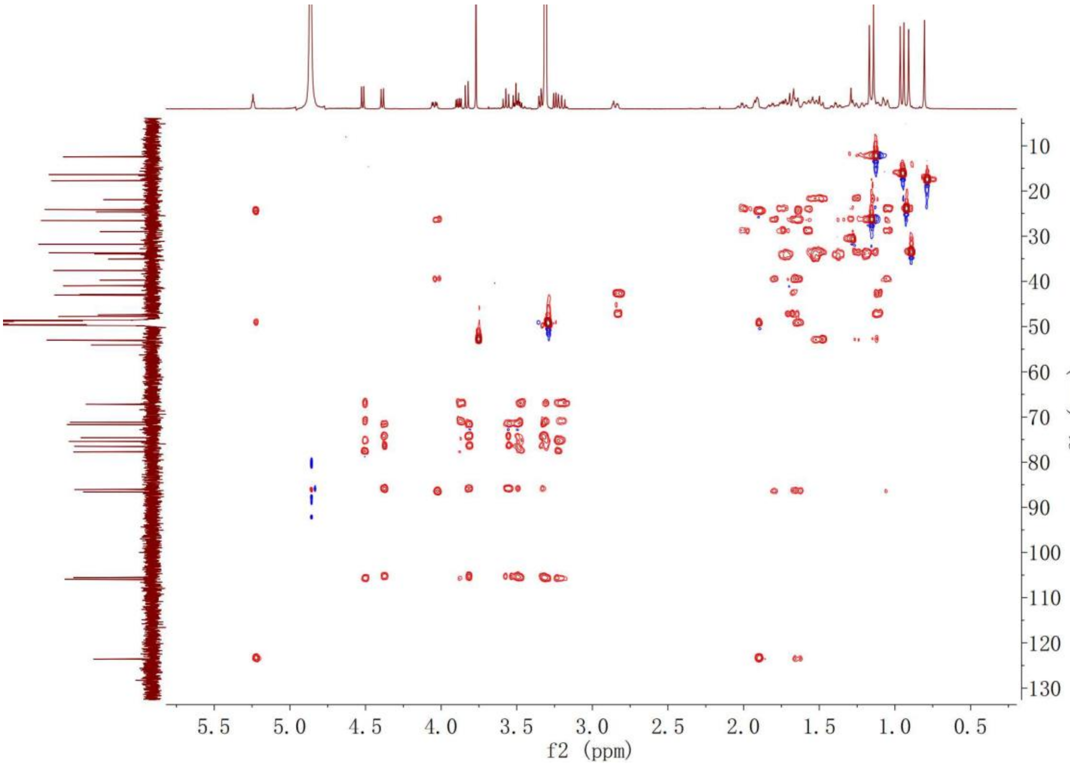

Figure S18. HSQC-TOCSY spectrum of compound **2**.

Formula Predictor Report - ZKF31.lcd Page 1 of 1

Data File: E:\DATA\2022\0518\ZKF31.lcd

Elmt	Val.	Min	Max	Elmt	Val.	Min	Max	Elmt	Val.	Min	Max	Elmt	Val.	Min	Max	Use Adduct
H	1	5	100	F	1	0	0	Cl	1	0	0	Ag	1	0	0	H
2H	1	0	0	Na	1	0	0	Co	2	0	0	I	3	0	0	
B	3	0	0	Mg	2	0	0	Cu	2	0	0	Ir	3	0	0	
C	4	10	50	Si	4	0	0	Se	2	0	0					
N	3	0	20	P	3	0	0	Br	1	0	0					
O	2	0	30	S	2	0	0	Pd	2	0	0					

Error Margin (ppm): 58
HC Ratio: unlimited
Max Isotopes: all
MSn Iso RI (%): 75.00

DBE Range: not fixed
Apply N Rule: no
Isotope RI (%): 1.00
MSn Logic Mode: OR

Electron Ions: both
Use MSn Info: yes
Isotope Res: 10000
Max Results: 30

Event#: 2 MS(E-) Ret. Time : 0.400 -> 0.440 Scan# : 62 -> 68

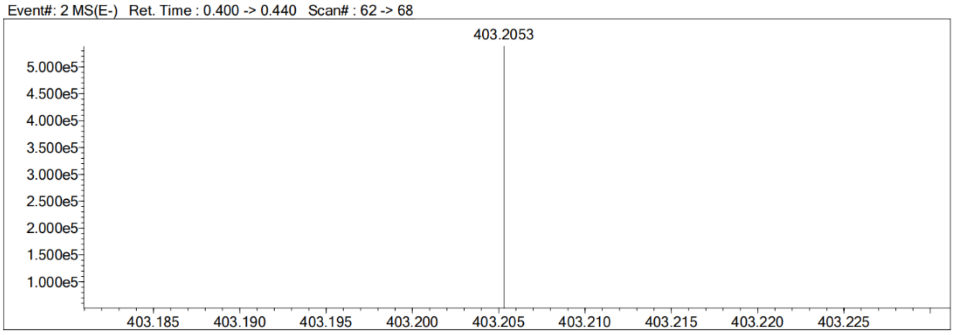

Measured region for 403.2053 m/z

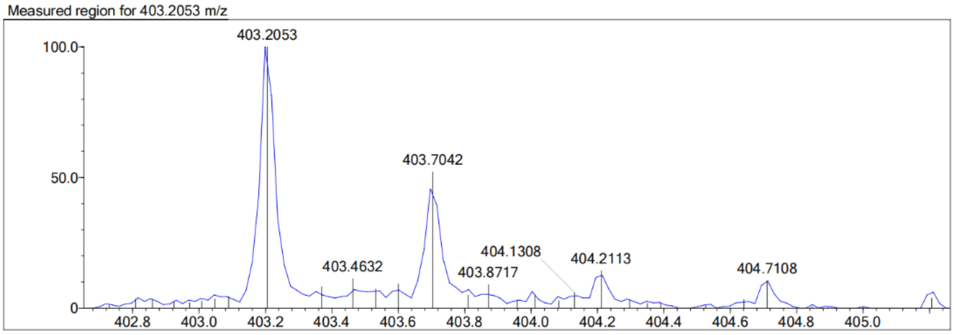

C42 H64 O15 [M-2H]2- : Predicted region for 403.2050 m/z

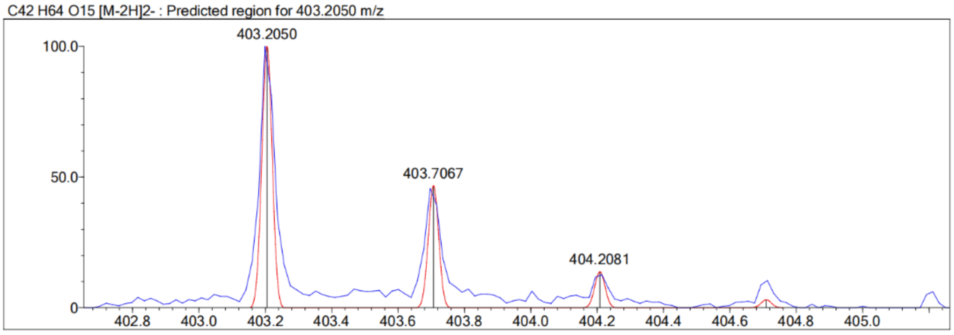

Formula (M)	Ion	Meas. m/z	Pred. m/z	Df. (mDa)	Df. (ppm)	DBE
C42 H64 O15	[M-2H]2-	403.2053	403.2050	0.3	0.74	11.0

Figure S19. HRESI-MS spectra of compound **2**.

Rudolph Research Analytical

This sample was measured on an Autopol VI, Serial #91058
Manufactured by Rudolph Research Analytical, Hackettstown, NJ, USA.

Measurement Date : Tuesday, 31-MAY-2022

Set Temperature : OFF

Time Delay : Disabled

Delay between Measurement : Disabled

n	Average	Std.Dev.	% RSD	Maximum	Minimum
5	4.31	0.59	13.68	5.06	3.48

S.No	Sample ID	Time	Result	Scale	OR °Arc	WLG.nm	Lg.mm	Conc.g/100ml	Temp.
1	ZKF-31	07:03:52 PM	5.06	SR	0.0045	589	100.00	0.089	25.2
2	ZKF-31	07:04:01 PM	4.04	SR	0.0036	589	100.00	0.089	25.2
3	ZKF-31	07:04:09 PM	4.49	SR	0.0040	589	100.00	0.089	25.2
4	ZKF-31	07:04:17 PM	4.49	SR	0.0040	589	100.00	0.089	25.1
5	ZKF-31	07:04:25 PM	3.48	SR	0.0031	589	100.00	0.089	25.1

Figure S20. Optical rotations spectrum of compound **2**.

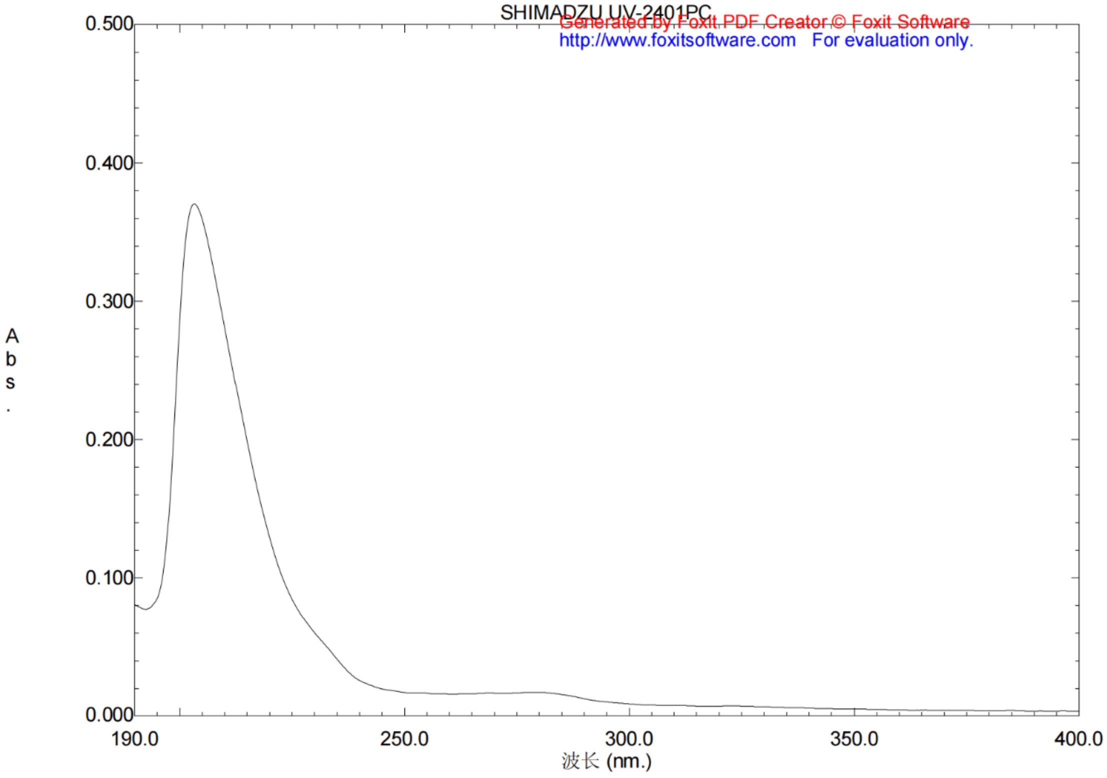

文件名: ZKF-31

ZKF-31 ———

样品浓度：0.0356毫克/毫升
溶剂：甲醇

创建于: 20:23 22-06-01
数据: 原始

测量模式: Abs.
扫描速度: 中速
狭缝: 5.0
采样间隔: 0.2

否.	波长 (nm.)	Abs.
1	279.40	0.0169
2	203.20	0.3704

Figure S21. UV spectrum of compound **2**.

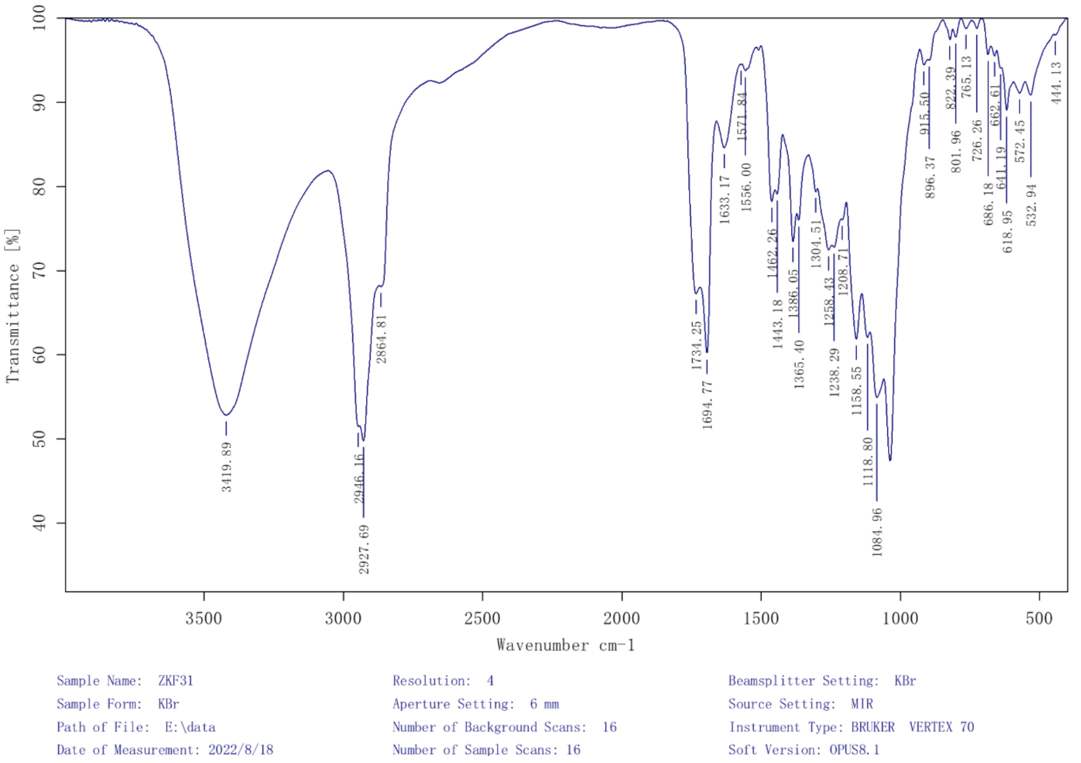

Figure S22. IR spectrum of compound **2**.

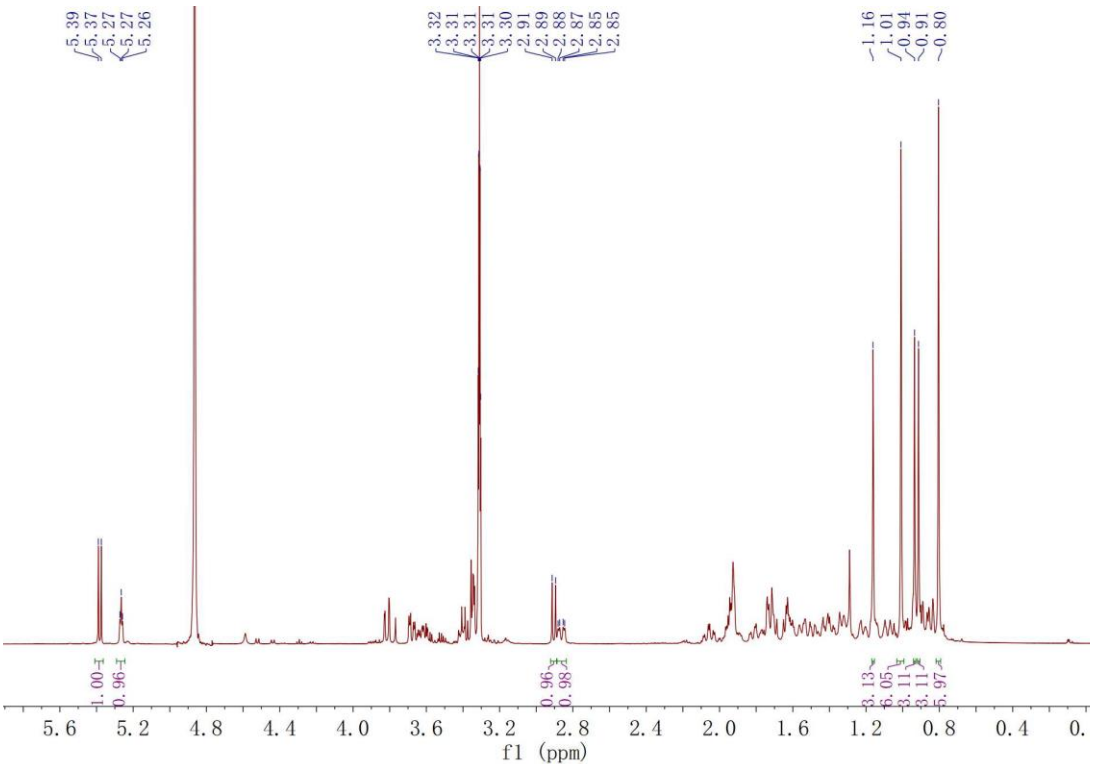

Figure S23. ^{1}H NMR spectrum of compound **3** (methanol-d_4, 500MHz).

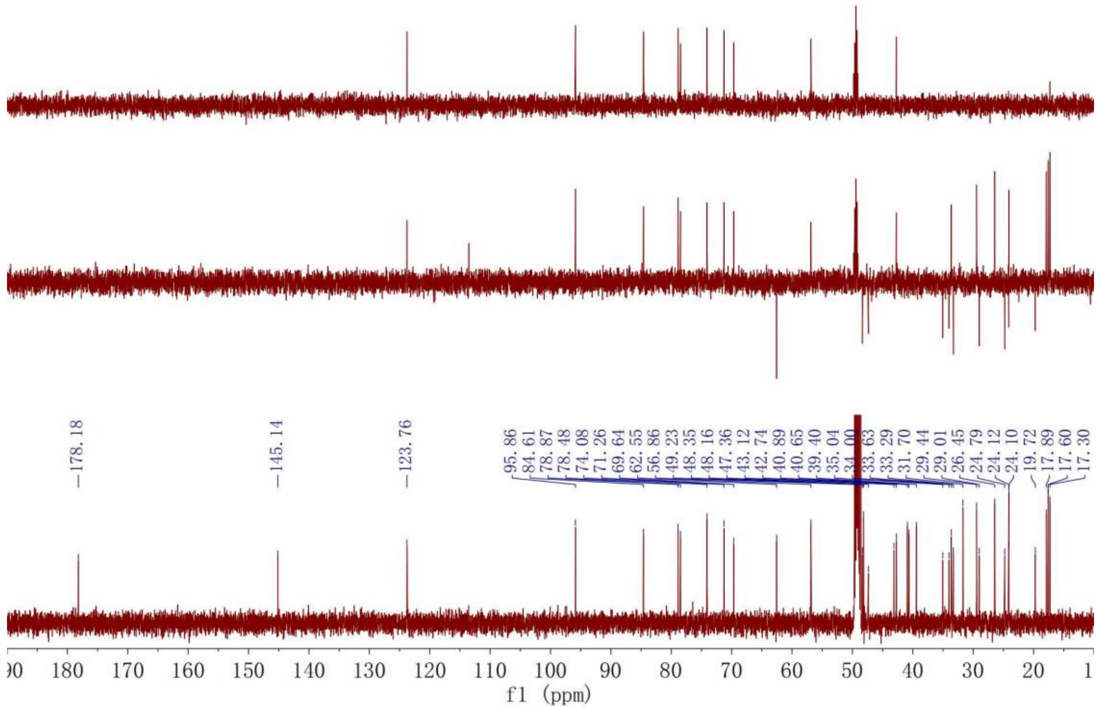

Figure S24. ^{13}C NMR spectrum of compound **3** (methanol-d_4, 125MHz).

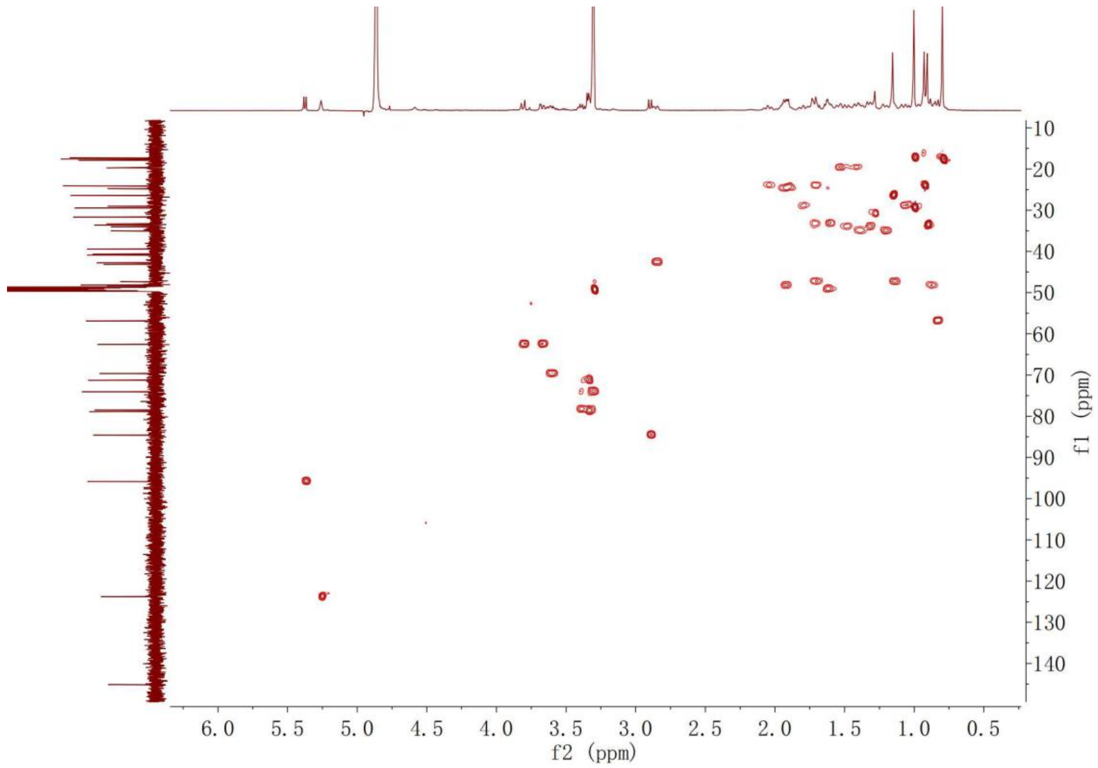

Figure S25. HSQC spectrum of compound **3.**

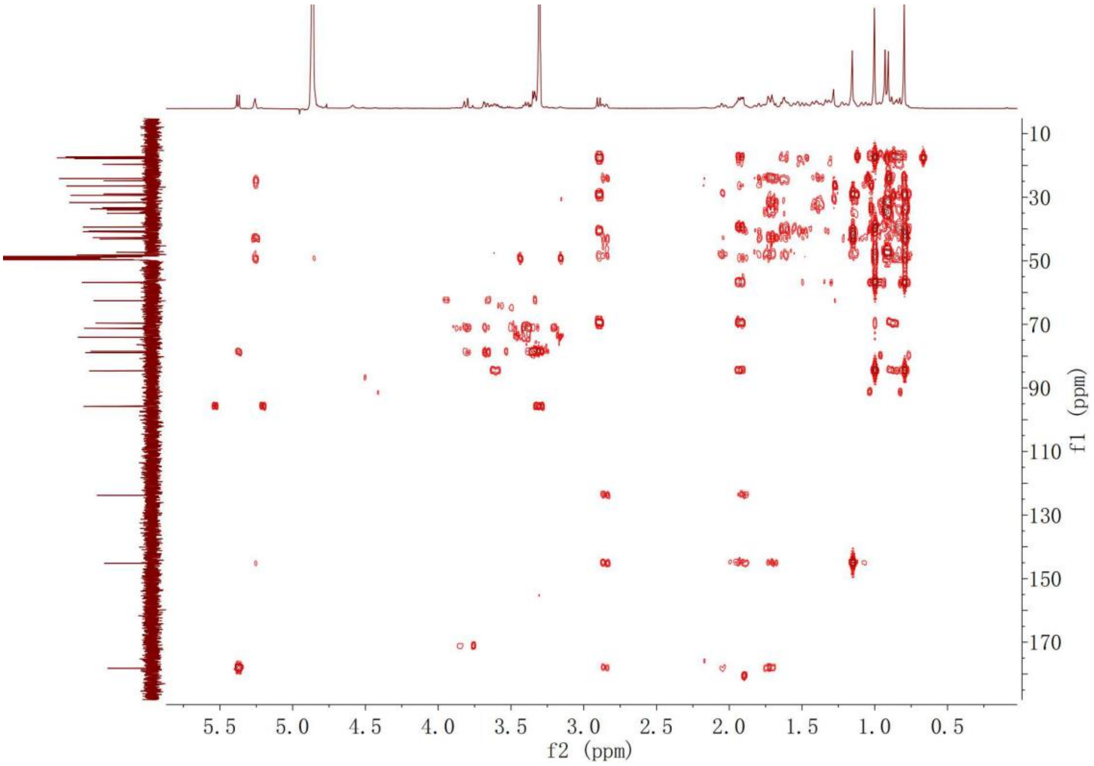

Figure S26. HMBC spectrum of compound **3.**

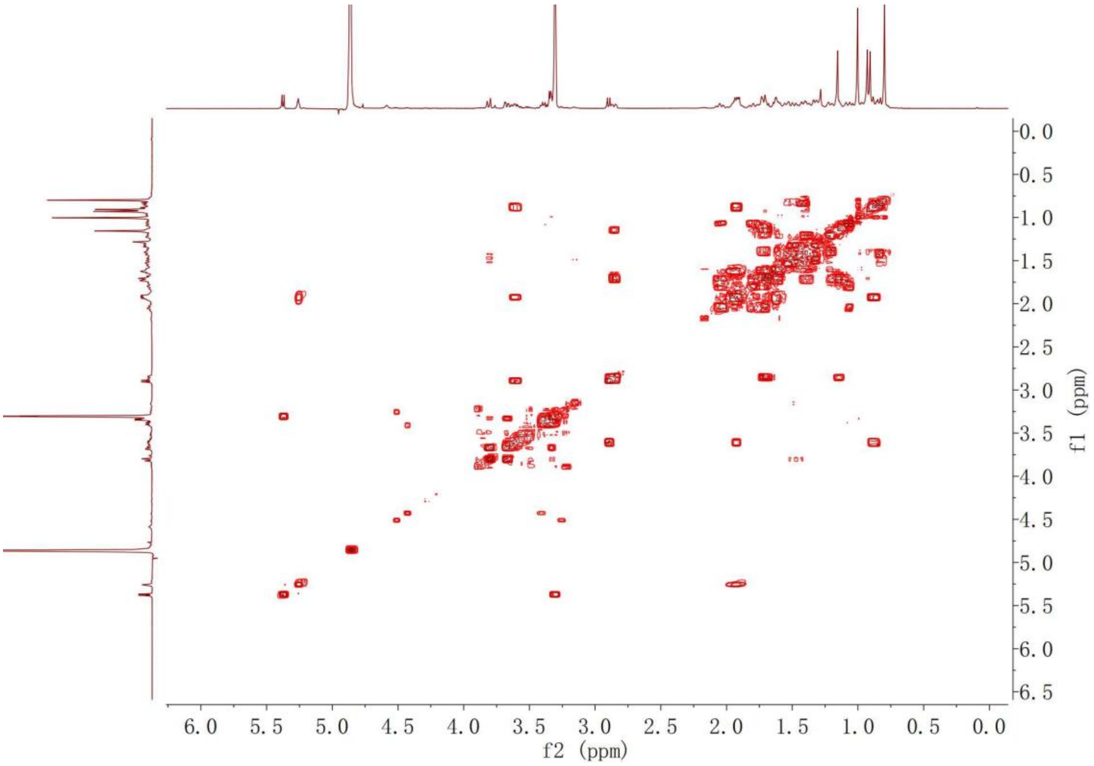

Figure S27. ^{1}H-^{1}H COSY spectrum of compound **3.**

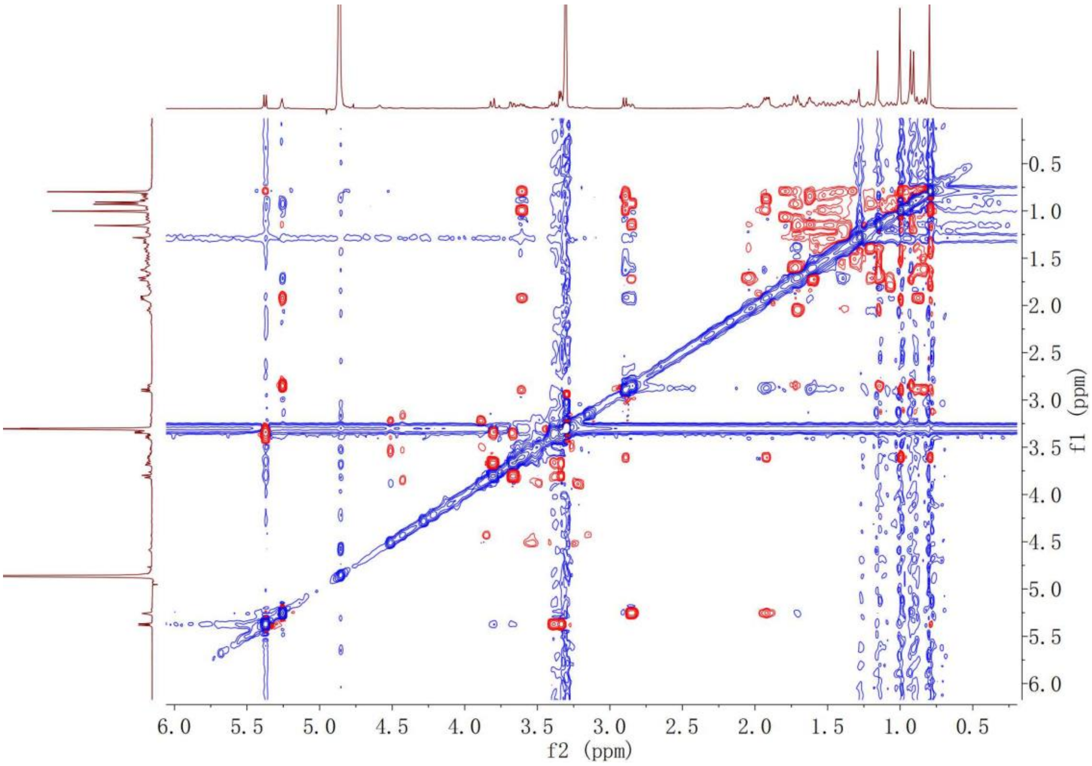

Figure S28. ROESY spectrum of compound **3**.

Formula Predictor Report - ZKF17.lcd Page 1 of 1

Data File: E:\DATA\2022\1021\1\ZKF17.lcd

Elmt	Val.	Min	Max	Elmt	Val.	Min	Max	Elmt	Val.	Min	Max	Elmt	Val.	Min	Max	Use Adduct
H	1	5	100	F	1	0	0	Cl	1	0	0	Ag	1	0	0	H
2H	1	0	0	Na	1	0	0	Co	2	0	0	I	3	0	0	Na
B	3	0	0	Mg	2	0	0	Cu	2	0	0	Ir	3	0	0	
C	4	10	60	Si	4	0	0	Se	2	0	0					
N	3	0	10	P	3	0	0	Br	1	0	0					
O	2	0	30	S	2	0	0	Pd	2	0	0					

Error Margin (ppm): 5
HC Ratio: unlimited
Max Isotopes: all
MSn Iso RI (%): 75.00

DBE Range: not fixed
Apply N Rule: no
Isotope RI (%): 1.00
MSn Logic Mode: OR

Electron Ions: both
Use MSn Info: yes
Isotope Res: 10000
Max Results: 30

Event#: 1 MS(E+) Ret. Time : 0.187 -> 0.787 Scan# : 29 -> 119

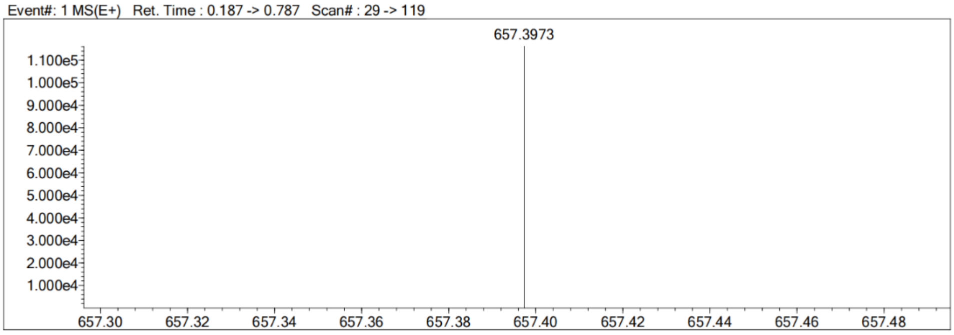

Measured region for 657.3973 m/z

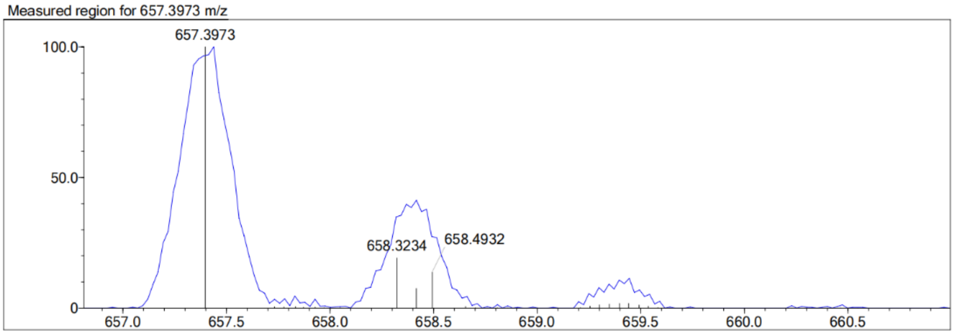

C36 H58 O9 [M+Na]+ : Predicted region for 657.3973 m/z

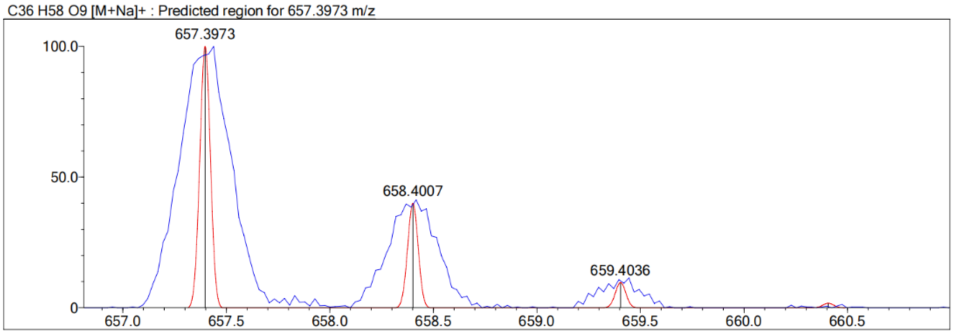

Formula (M)	Ion	Meas. m/z	Pred. m/z	Df. (mDa)	Df. (ppm)	DBE
C36 H58 O9	[M+Na]+	657.3973	657.3973	-0.0	0.00	8.0

Figure S29. HRESI-MS spectra of compound **3**.

Rudolph Research Analytical

This sample was measured on an Autopol VI, Serial #91058
Manufactured by Rudolph Research Analytical, Hackettstown, NJ, USA.

Measurement Date : Monday, 17-OCT-2022

Set Temperature : OFF

Time Delay : Disabled

Delay between Measurement : Disabled

n	Average	Std.Dev.	% RSD	Maximum	Minimum
5	23.14	0.64	2.76	23.84	22.67

S.No	Sample ID	Time	Result	Scale	OR °Arc	WLG.nm	Lg.mm	Conc.g/100ml	Temp.
1	ZKF17	07:15:53 PM	23.84	SR	0.041	589	100.00	0.172	18.6
2	ZKF17	07:15:59 PM	22.67	SR	0.039	589	100.00	0.172	18.6
3	ZKF17	07:16:06 PM	22.67	SR	0.039	589	100.00	0.172	18.6
4	ZKF17	07:16:12 PM	23.84	SR	0.041	589	100.00	0.172	18.6
5	ZKF17	07:16:18 PM	22.67	SR	0.039	589	100.00	0.172	18.5

Figure S30. Optical rotations spectrum of compound **3**.

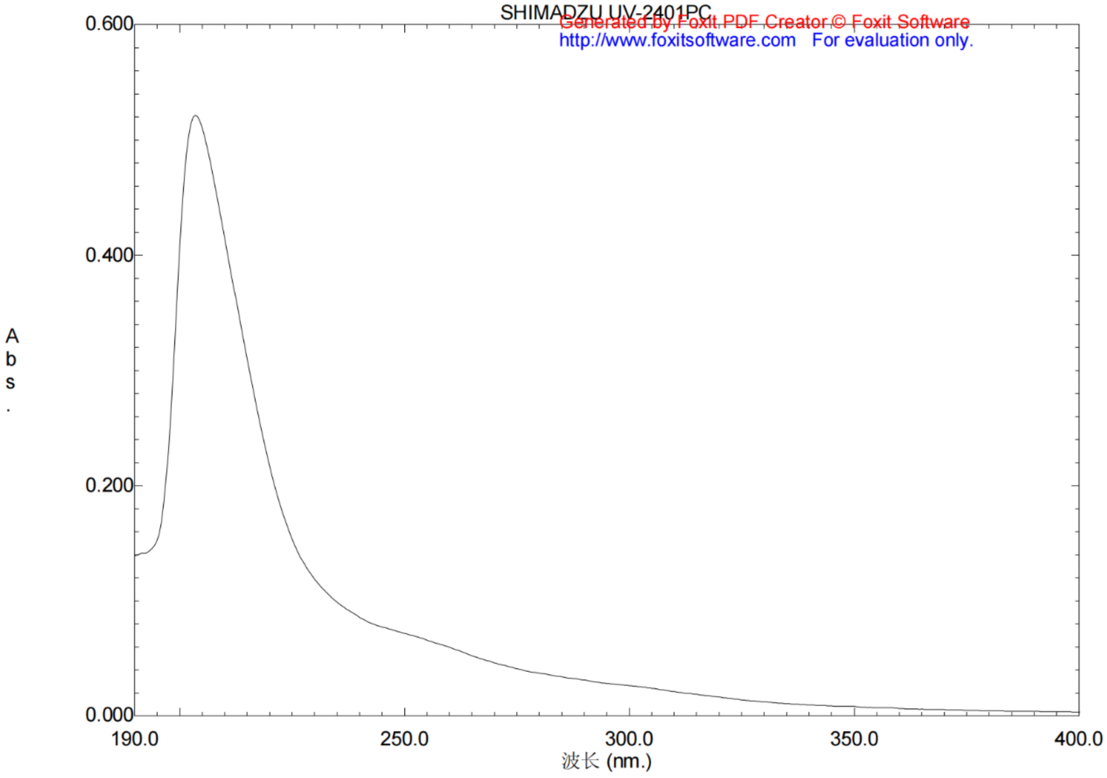

文件名: ZKF17

ZKF17 ———

创建于: 14:44 22-10-20
数据: 原始

样品浓度：0.0573毫克/毫升
溶剂：甲醇

测量模式: Abs.
扫描速度: 中速
狭缝: 5.0
采样间隔: 0.5

否.	波长 (nm.)	Abs.
1	203.50	0.5213

Figure S31. UV spectrum of compound **3**.

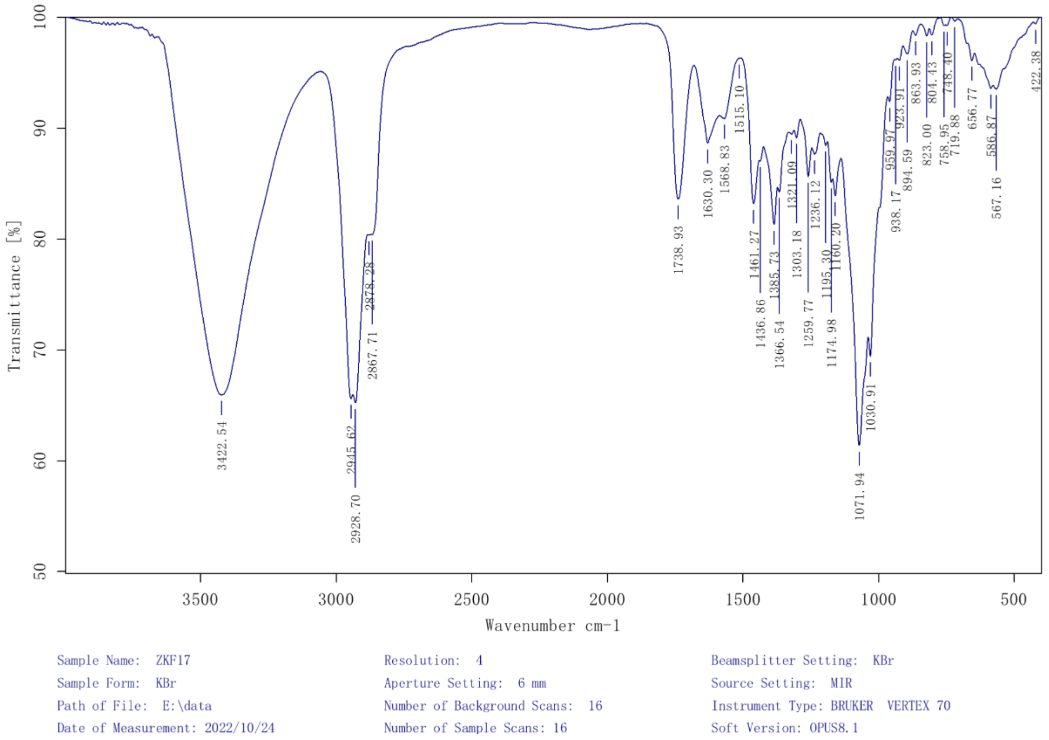

Figure S32. IR spectrum of compound **3**.

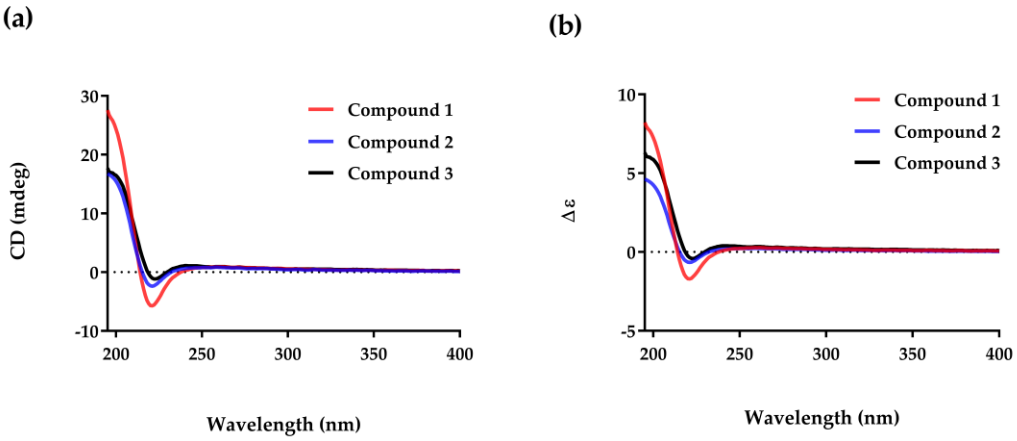

Figure S33. The CD spectra of compounds **1**, **2**, and **3** in MeOH.

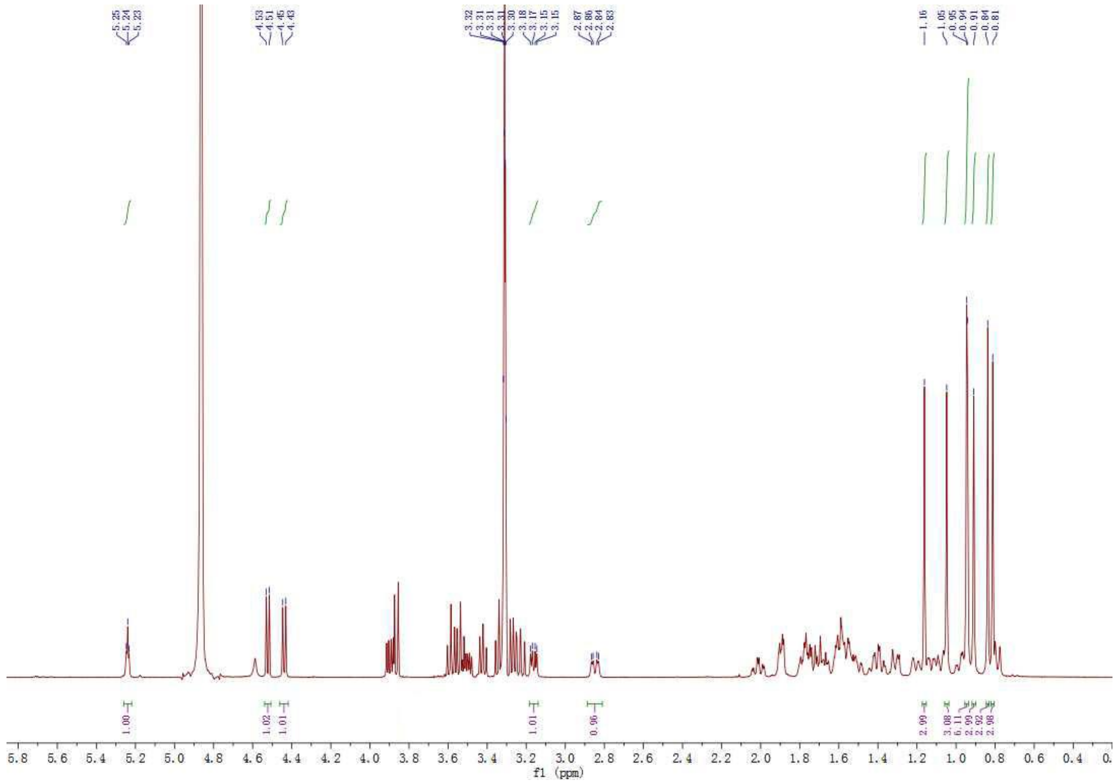

Figure S34. ^{1}H NMR spectrum of compound **4** (methanol-d_4, 500MHz).

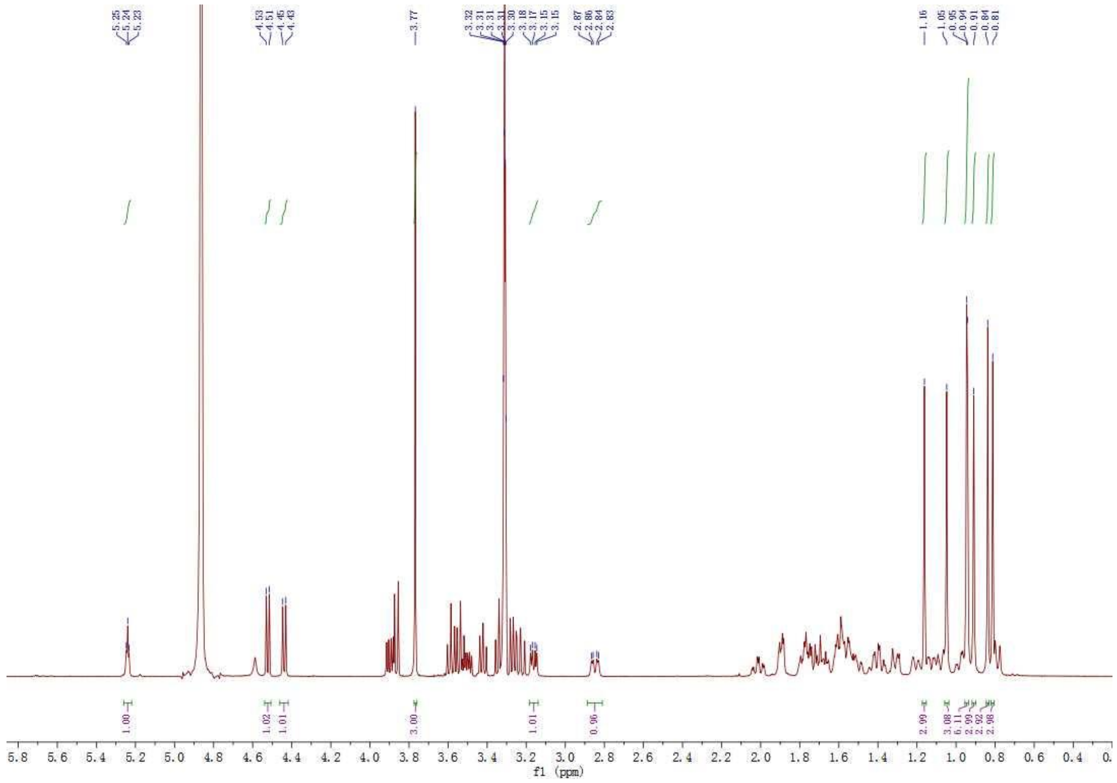

Figure S35. ^{1}H NMR spectrum of compound **5** (methanol-d_4, 500MHz).

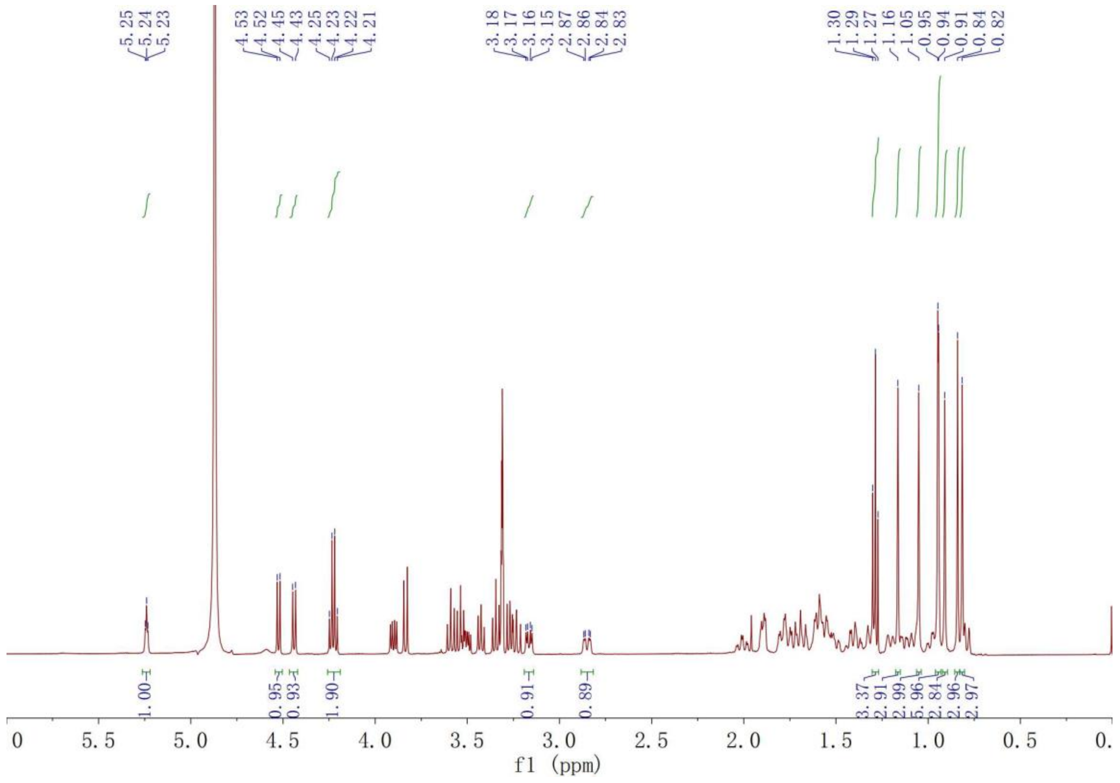

Figure S36. ^{1}H NMR spectrum of compound **6** (methanol-d_4, 500MHz).

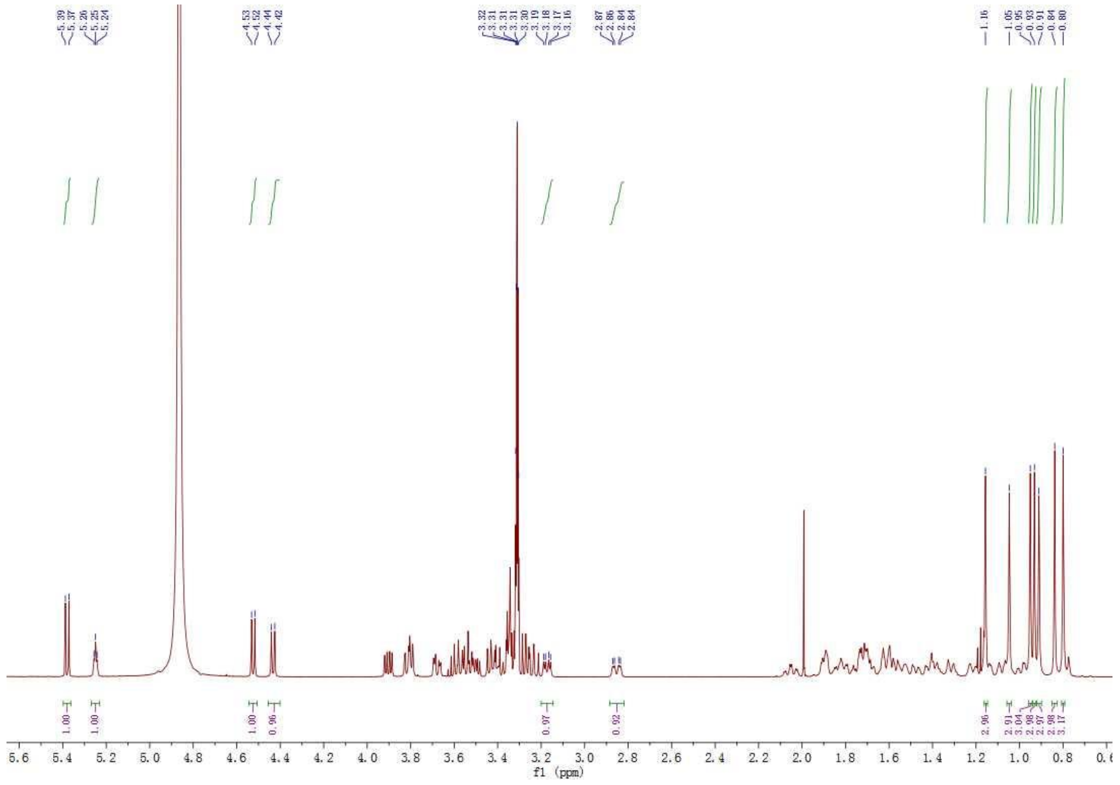

Figure S37. ^{1}H NMR spectrum of compound **7** (methanol-d_4, 500MHz).

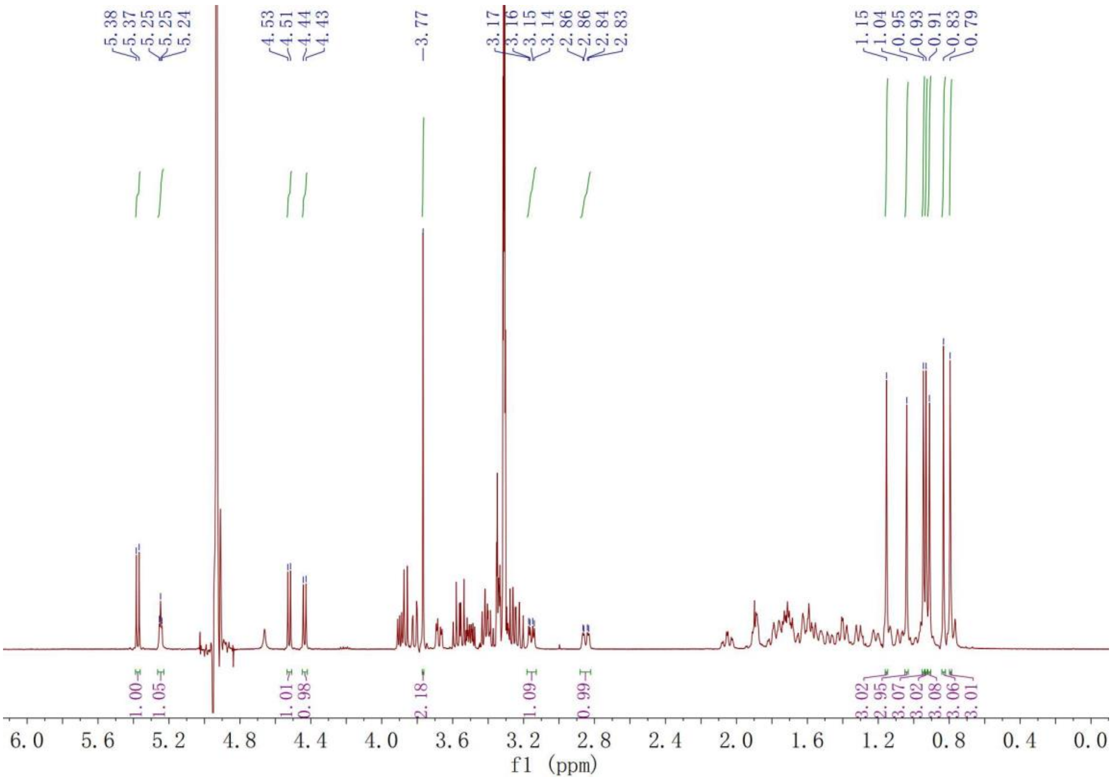

Figure S38. ^{1}H NMR spectrum of compound **8** (methanol-*d*4, 500MHz).

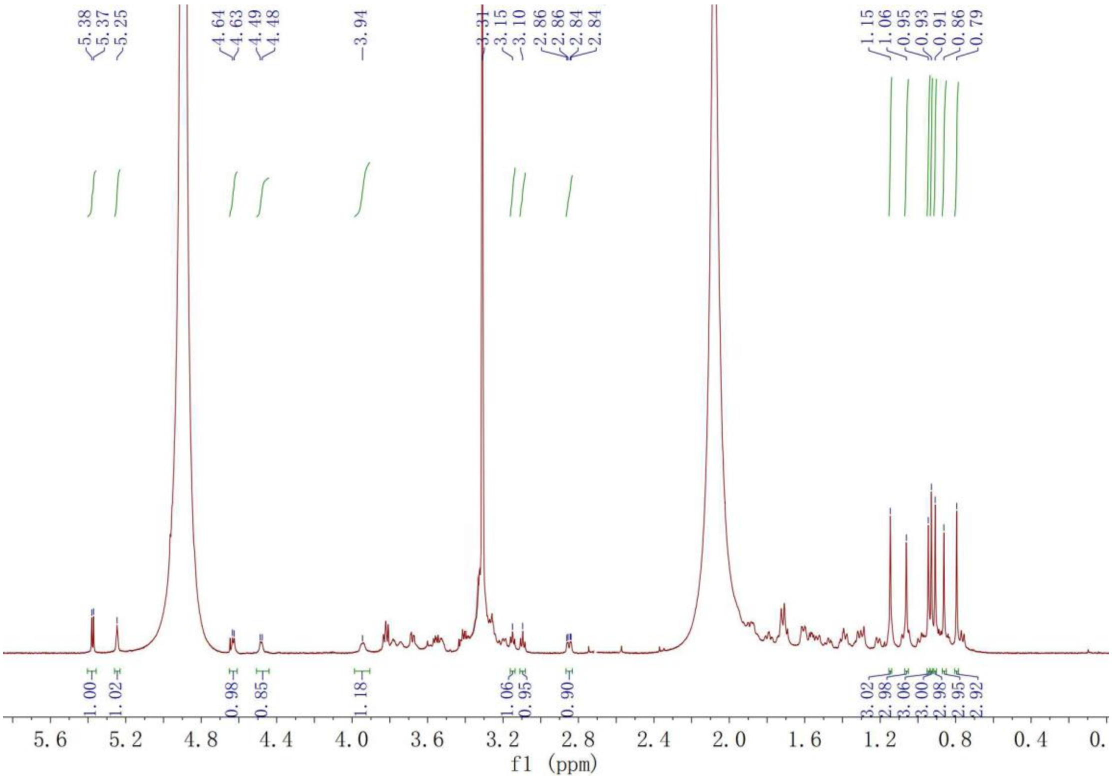

Figure S39. ^{1}H NMR spectrum of compound **9** (methanol-*d*4, 500MHz).

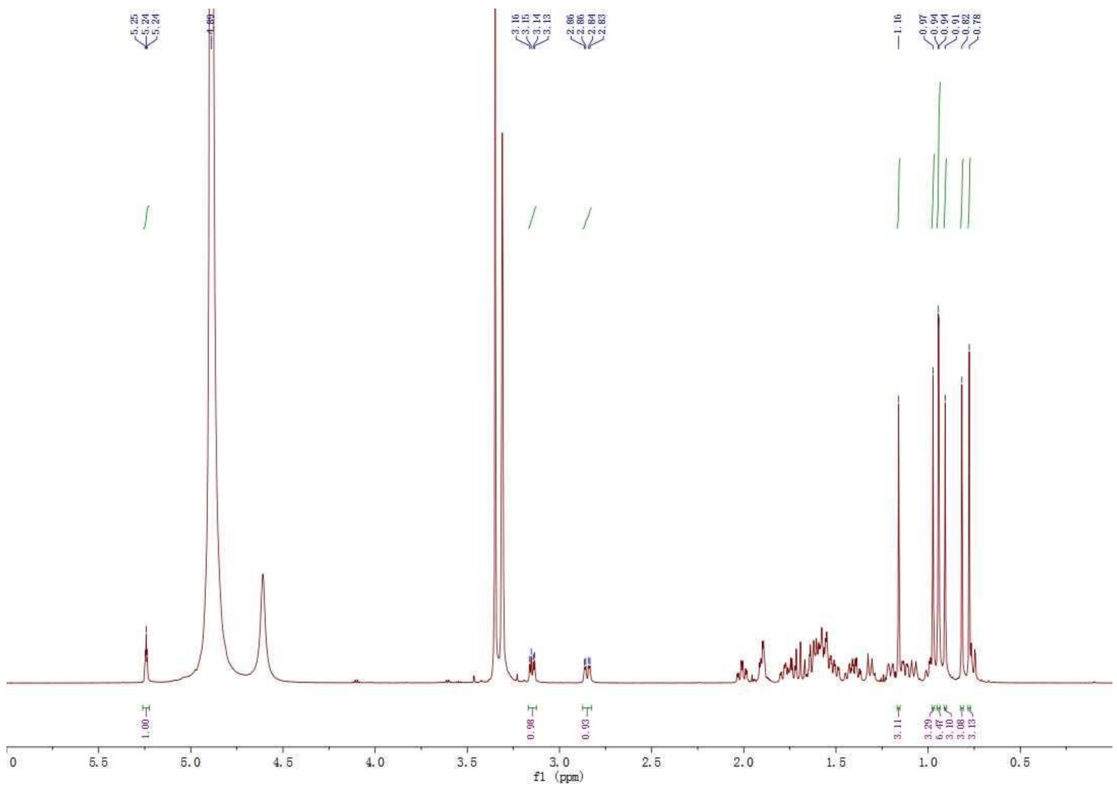

Figure S40. ^{1}H NMR spectrum of compound **10** (methanol-d_4, 600MHz).

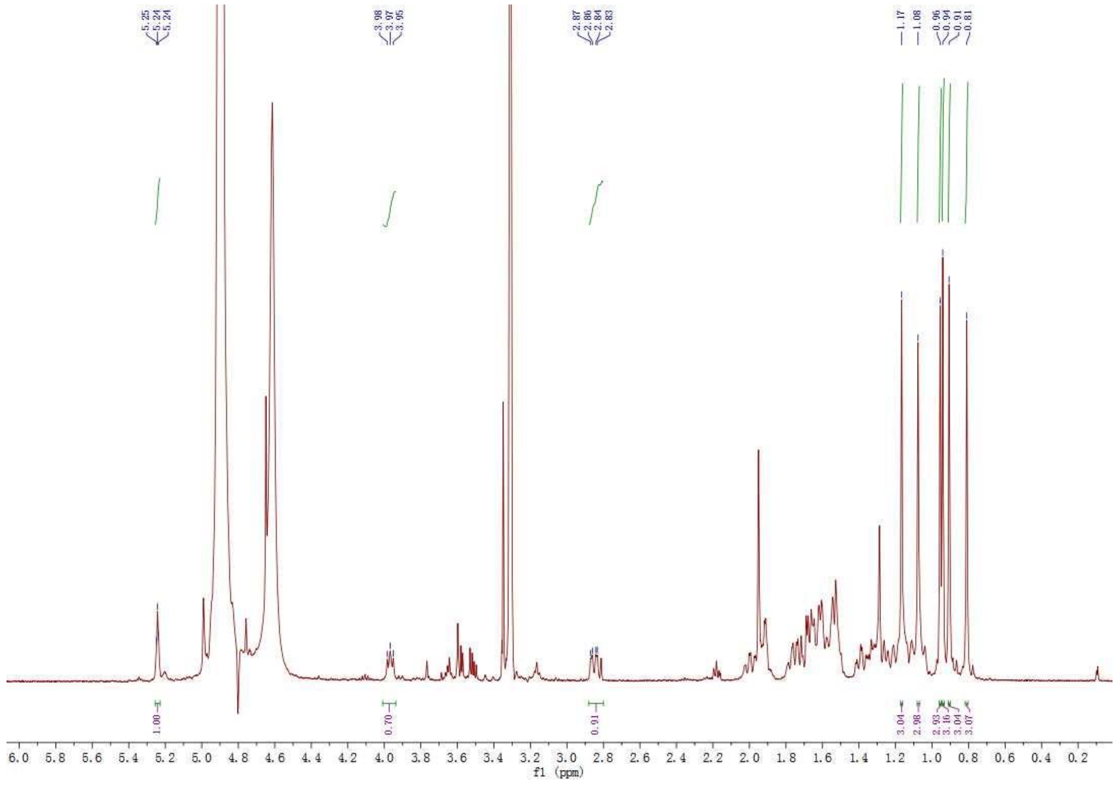

Figure S41. ^{1}H NMR spectrum of compound **11** (methanol-d_4, 500MHz).

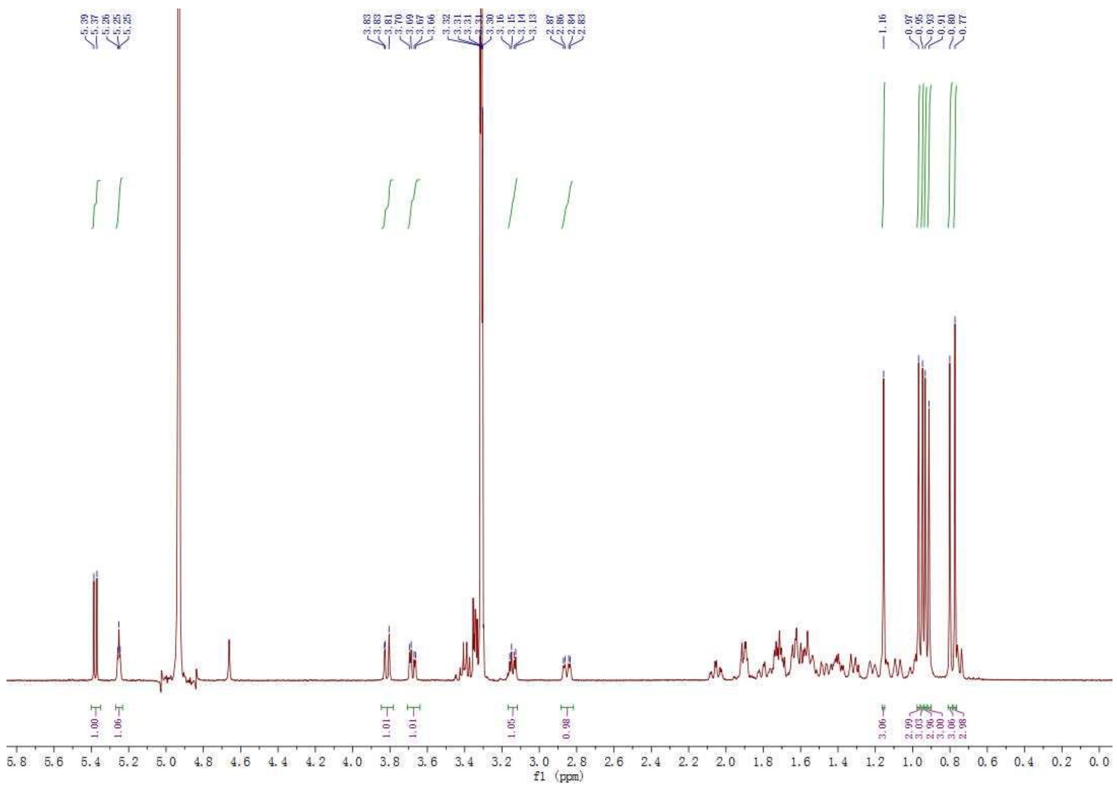

Figure S42. ^{1}H NMR spectrum of compound **12** (methanol-*d*4, 500MHz).

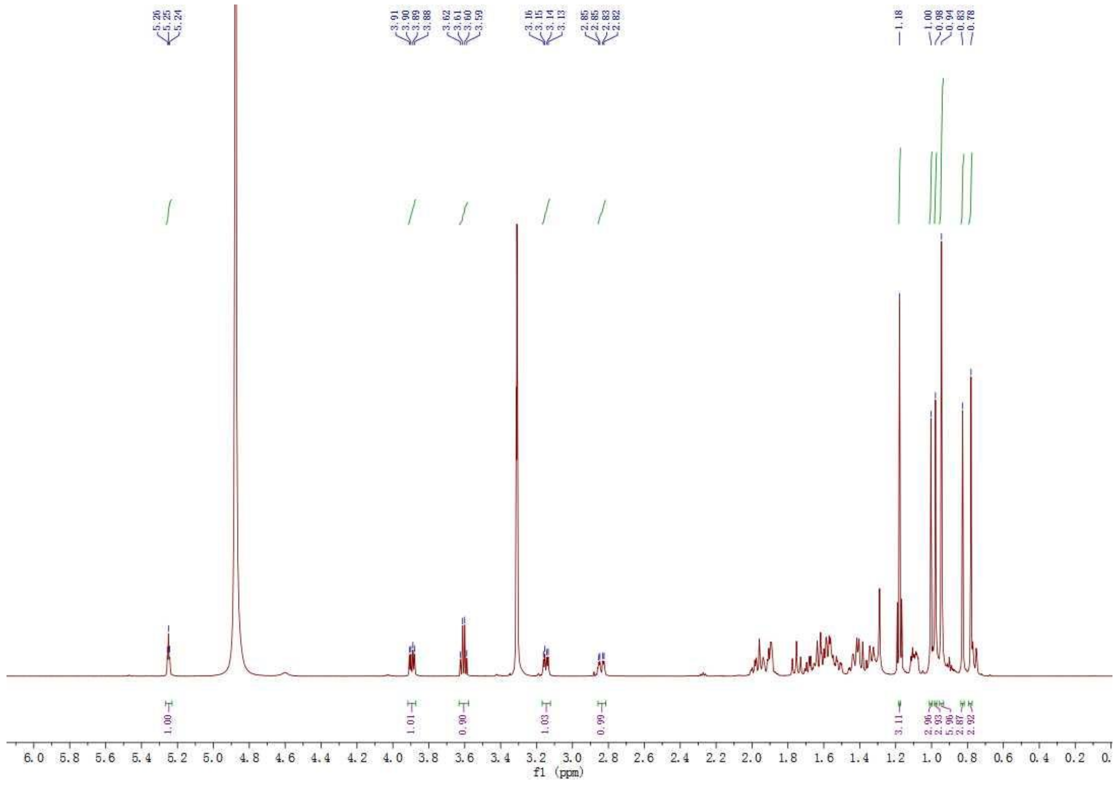

Figure S43. ^{1}H NMR spectrum of compound **13** (methanol-*d*4, 600MHz).

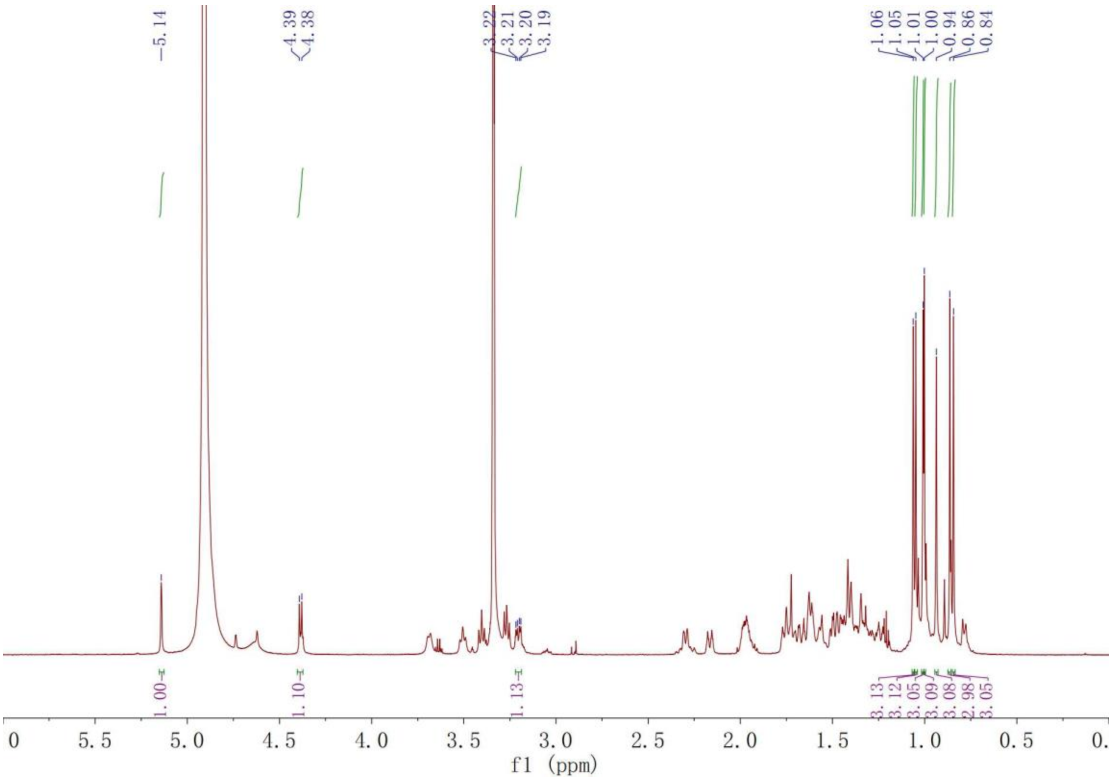

Figure S44. ^{1}H NMR spectrum of compound **14** (methanol-d_4, 600MHz).

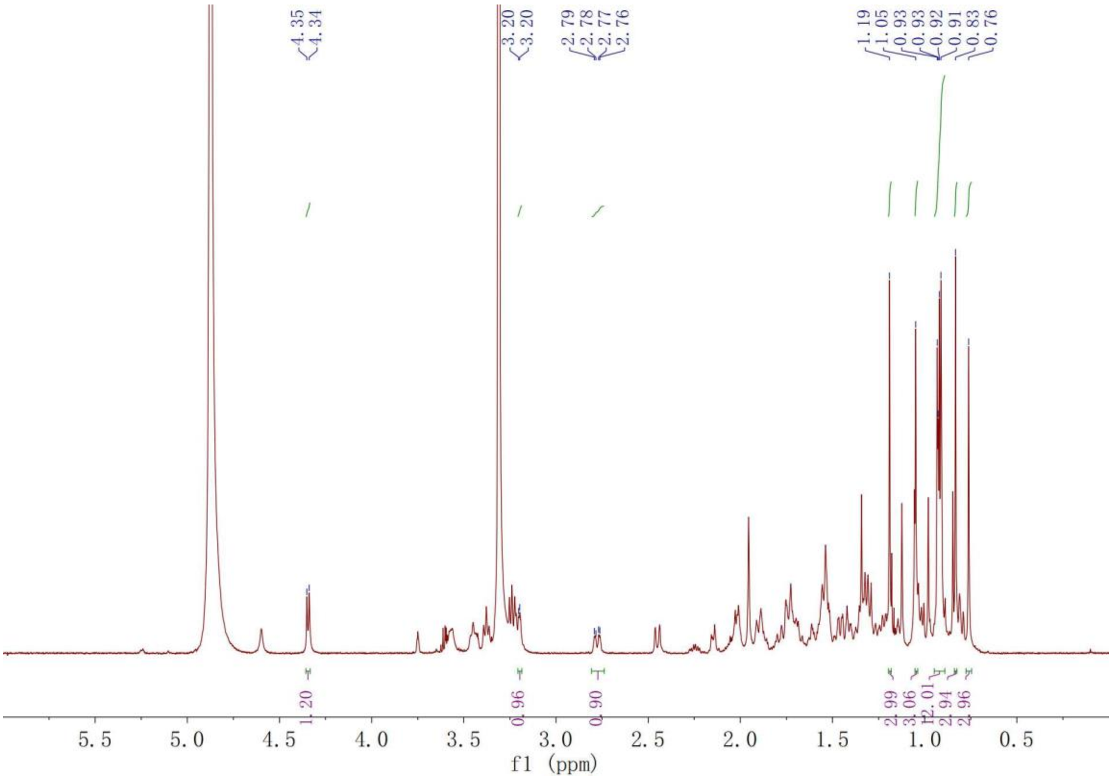

Figure S45. ^{1}H NMR spectrum of compound **15** (methanol-d_4, 600MHz).

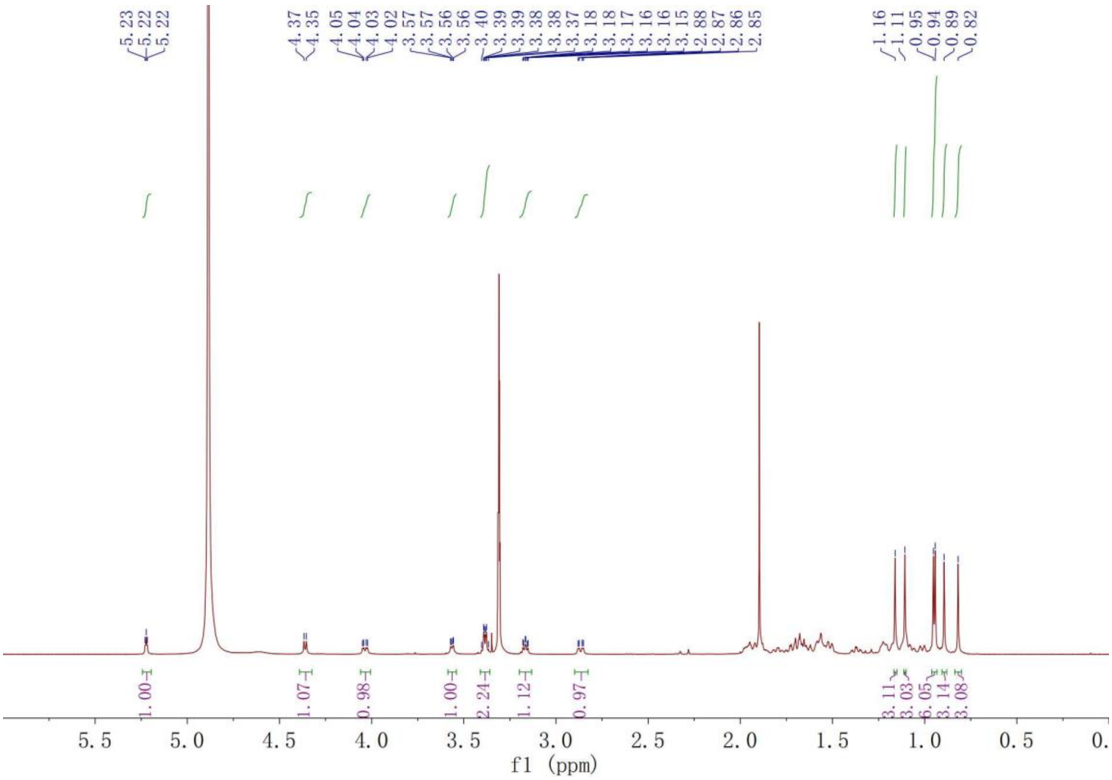

Figure S46. ^{1}H NMR spectrum of compound **16** (methanol-d_4, 600MHz).

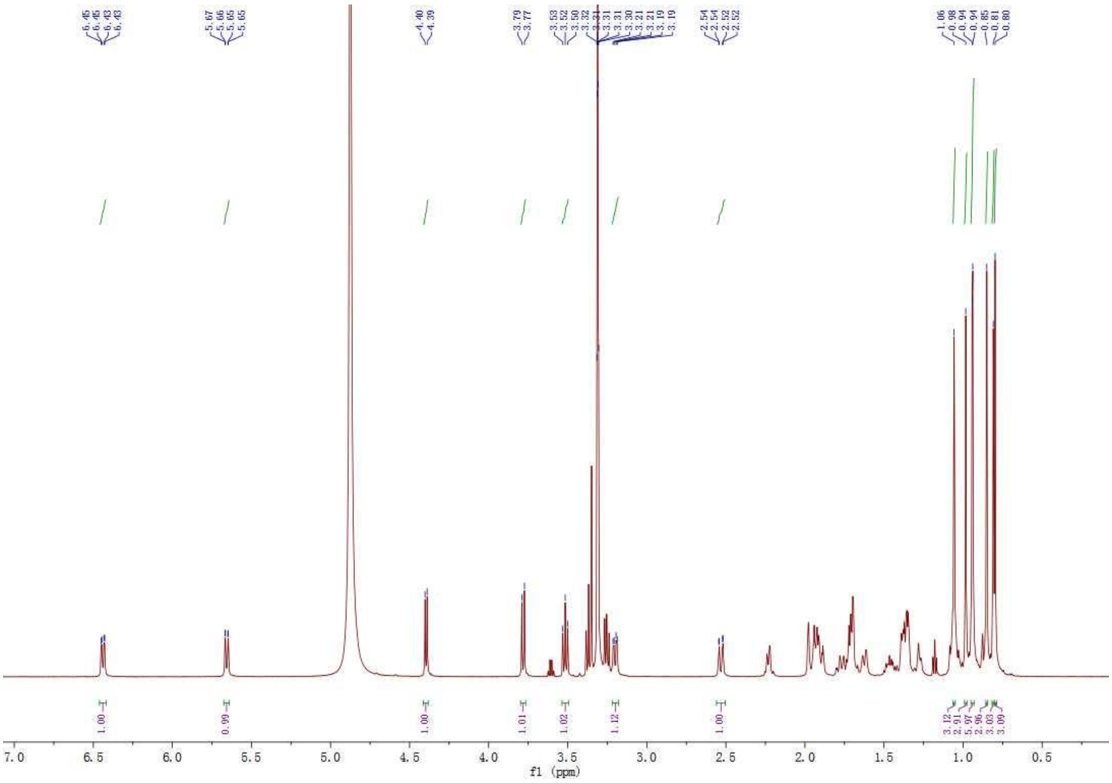

Figure S47. ^{1}H NMR spectrum of compound **17** (methanol-d_4, 600MHz).

Table S1. The data that was used to calculate the IC_{50} value for compound **3**.

Concentration (μM)	The absorbance was determined at 405 nm		
0	1.8958	1.9019	1.8899
100	0.6803	0.6919	0.7170
50	0.7423	0.7590	0.7817
25	0.8752	0.8998	0.8730
12.5	0.9834	0.9814	1.0439
6.25	1.2902	1.2382	1.2113
3.125	1.4815	1.5014	1.4437
1.5625	1.6101	1.5873	1.5432

Table S2. The data that was used to calculate the IC_{50} value for compound **9**.

Concentration (μM)	The absorbance was determined at 405 nm		
0	1.8958	1.9019	1.8899
100	0.4444	0.4341	0.4423
50	0.4621	0.4971	0.4936
25	0.6253	0.6273	0.6271
12.5	0.7563	0.7374	0.7484
6.25	0.8887	0.9370	0.9375
3.125	1.0034	0.9730	0.9284
1.5625	1.0695	1.1973	1.1694

Table S3. The data that was used to calculate the IC_{50} value for compound **13**.

Concentration (μM)	The absorbance was determined at 405 nm		
0	1.3835	1.3330	1.4915
50	0.4252	0.4282	0.4126
25	0.4835	0.5313	0.4158
12.5	0.6657	0.7189	0.6798
6.25	1.1589	1.1940	1.2485
3.125	1.3230	1.3611	1.4017
1.5625	1.3461	1.3679	1.4208
0.78125	1.3527	1.3974	1.4329

Table S4. The data that was used to calculate the IC_{50} value for acarbose.

Concentration (μM)	The absorbance was determined at 405 nm		
0	2.1073	2.1278	2.1184
0.012207031	0.2813	0.2694	0.2728

0.006103516	0.4321	0.4114	0.3998
0.003051758	0.6511	0.6945	0.6400
0.001525879	0.9629	0.9268	0.8430
0.000762939	0.8613	0.7828	0.7353
0.00038147	1.3322	1.4116	1.2263
0.000190735	1.3945	1.5378	1.3163

Table S5. The data that was used to calculate the Km, K_i, *and* K_i' value for compound **3**.

Inhibitor concentration (µM), Substrate concentration (µM)	At 40 min, the absorbance at 405 nm			At 60 min, the absorbance at 405 nm		
0, 20	2.389	2.3089	2.3749	3.7430	3.7856	3.7507
0, 10	1.9815	2.0409	2.0955	3.6091	3.6436	3.6622
0, 5	1.4382	1.5183	1.5654	2.8114	2.8911	2.9381
0, 2.5	0.9304	0.9691	0.9855	1.7553	1.8237	1.8345
0, 1.25	0.4709	0.5034	0.5031	1.0150	1.0704	1.0237
10, 20	1.7721	1.7106	1.6819	3.0562	3.0062	2.9897
10, 10	1.3527	1.3746	1.3316	2.6308	2.6593	2.5884
10, 5	0.8755	0.9599	0.8015	1.7481	1.9158	1.4774
10, 2.5	0.6534	0.6553	0.6477	1.3342	1.3436	1.3216
10, 1.25	0.4508	0.4348	0.4538	0.8708	0.8533	0.8824
20, 20	1.3537	1.4013	1.4327	2.5861	2.6594	2.6711
20, 10	1.1342	1.1695	1.1576	2.2100	2.2641	2.2381
20, 5	0.9566	0.9016	0.8443	1.7477	1.7496	1.6806
20, 2.5	0.5977	0.5982	0.6017	1.1970	1.2155	1.1990
20, 1.25	0.4184	0.4411	0.4376	0.8111	0.8186	0.8069
30, 20	1.3338	1.2450	1.3039	2.5147	2.4105	2.4819
30, 10	1.0438	1.0267	1.0430	2.0192	2.0074	2.0523
30, 5	0.8664	0.8472	0.8355	1.6416	1.6235	1.6226
30, 2.5	0.5481	0.5671	0.5655	1.0793	1.0715	1.1152
30, 1.25	0.3996	0.3989	0.3910	0.7365	0.7384	0.7435

Table S6. The data that was used to calculate the Km, K_i, *and* K_i' value for compound **9**.

Inhibitor concentration (µM), Substrate concentration (µM)	At 40 min, the absorbance at 405 nm			At 60 min, the absorbance at 405 nm		
0, 20	1.7770	1.7276	1.6815	3.1858	3.1595	3.1413
0, 10	1.2788	1.2958	1.2796	2.7135	2.7351	2.7318
0, 5	0.9435	0.9631	0.9686	2.0897	2.1274	2.1005
0, 2.5	0.6044	0.6198	0.6389	1.3440	1.397	1.4151

0, 1.25	0.4016	0.4044	0.4167	0.8927	0.8869	0.9061
1, 20	1.1836	1.1608	1.1294	2.5202	2.4940	2.4150
1, 10	0.9300	0.9261	0.8717	2.0569	2.0714	1.9441
1, 5	0.7181	0.6615	0.6738	1.6108	1.4904	1.5321
1, 2.5	0.5198	0.4789	0.4803	1.1546	1.0906	1.0878
1, 1.25	0.3628	0.3418	0.3450	0.7934	0.7671	0.765
15, 20	0.6873	0.6806	0.6860	1.5269	1.5028	1.5141
15, 10	0.5631	0.5584	0.5762	1.2792	1.2649	1.3213
15, 5	0.4552	0.4583	0.4585	1.0338	1.045	1.0553
15, 2.5	0.3533	0.3457	0.3541	0.7855	0.7751	0.8129
15, 1.25	0.2585	0.2579	0.2520	0.5664	0.5730	0.5667
30, 20	0.5788	0.5855	0.5712	1.2477	1.2704	1.2314
30, 10	0.4741	0.4720	0.4750	1.0277	1.0346	1.0411
30, 5	0.3895	0.3841	0.3769	0.8658	0.8555	0.8353
30, 2.5	0.2916	0.2954	0.2993	0.6373	0.6469	0.6562
30, 1.25	0.2282	0.2232	0.2260	0.4892	0.4743	0.4793

Table S7. The data that was used to calculate the Km, K_i*, and* K_i' value for compound **13**.

Inhibitor concentration (µM), Substrate concentration (µM)	At 40 min, the absorbance at 405 nm			At 42 min, the absorbance at 405 nm		
0, 20	0.8897	0.8690	0.8560	1.1424	1.1263	1.1087
0, 10	0.6616	0.6628	0.6686	0.8643	0.8834	0.8734
0, 5	0.4742	0.4664	0.4818	0.6329	0.6182	0.6489
0, 2.5	0.3250	0.3233	0.3241	0.4391	0.4361	0.4332
0, 1.25	0.2127	0.2137	0.2167	0.2775	0.2797	0.2780
5, 20	0.6681	0.6589	0.6944	0.8635	0.8479	0.8889
5, 10	0.4885	0.4832	0.5273	0.6347	0.6313	0.6835
5, 5	0.3071	0.3248	0.3327	0.4012	0.4300	0.4372
5, 2.5	0.2150	0.2199	0.2261	0.2822	0.2910	0.2993
5, 1.25	0.1505	0.1474	0.1521	0.1947	0.1927	0.1958
10, 20	0.4291	0.4068	0.4258	0.5371	0.5130	0.5350
10, 10	0.3402	0.3144	0.3391	0.4314	0.3998	0.4320
10, 5	0.2353	0.2200	0.2359	0.2985	0.2826	0.3006
10, 2.5	0.1573	0.1522	0.1615	0.2037	0.1937	0.2067
10, 1.25	0.1138	0.1106	0.1182	0.1411	0.1389	0.1479
20, 20	0.3343	0.3345	0.3517	0.4152	0.4173	0.4392
20, 10	0.2677	0.2821	0.2870	0.3361	0.3527	0.3592
20, 5	0.1987	0.1952	0.2055	0.2515	0.2454	0.2578
20, 2.5	0.1429	0.1453	0.1436	0.1821	0.1830	0.1798
20, 1.25	0.1093	0.1023	0.1057	0.1342	0.1252	0.1290
